# Protocol for the establishment of a serine integrase-based platform for functional validation of genetic switch controllers in eukaryotic cells

**DOI:** 10.1371/journal.pone.0303999

**Published:** 2024-05-23

**Authors:** Marco A. de Oliveira, Lilian H. Florentino, Thais T. Sales, Rayane N. Lima, Luciana R. C. Barros, Cintia G. Limia, Mariana S. M. Almeida, Maria L. Robledo, Leila M. G. Barros, Eduardo O. Melo, Daniela M. Bittencourt, Stevens K. Rehen, Martín H. Bonamino, Elibio Rech

**Affiliations:** 1 Department of Cell Biology, Institute of Biological Science, University of Brasília, Brasília, Distrito Federal, Brazil; 2 National Institute of Science and Technology in Synthetic Biology (INCT BioSyn), Brasília, Distrito Federal, Brazil; 3 Embrapa Genetic Resources and Biotechnology, Brasília, Distrito Federal, Brazil; 4 Center for Translational Research in Oncology, Instituto do Câncer do Estado de São Paulo, Hospital das Clínicas da Faculdade de Medicina de Universidade de São Paulo, São Paulo, Brazil; 5 Molecular Carcinogenesis Program, Research Coordination, National Cancer Institute (INCA), Rio de Janeiro, Brazil; 6 D’Or Institute for Research and Education (IDOR), Rio de Janeiro, Brazil; 7 Institute of Biomedical Sciences, Federal University of Rio de Janeiro, Rio de Janeiro, Brazil; 8 Cell and Gene Therapy Program, Research Coordination, National Cancer Institute (INCA), Rio de Janeiro, Brazil; 9 Vice-Presidency of Research and Biological Collections (VPPCB), FIOCRUZ – Oswaldo Cruz Foundation Institute, Rio de Janeiro, Brazil; Fudan University, CHINA

## Abstract

Serine integrases (Ints) are a family of site-specific recombinases (SSRs) encoded by some bacteriophages to integrate their genetic material into the genome of a host. Their ability to rearrange DNA sequences in different ways including inversion, excision, or insertion with no help from endogenous molecular machinery, confers important biotechnological value as genetic editing tools with high host plasticity. Despite advances in their use in prokaryotic cells, only a few Ints are currently used as gene editors in eukaryotes, partly due to the functional loss and cytotoxicity presented by some candidates in more complex organisms. To help expand the number of Ints available for the assembly of more complex multifunctional circuits in eukaryotic cells, this protocol describes a platform for the assembly and functional screening of serine-integrase-based genetic switches designed to control gene expression by directional inversions of DNA sequence orientation. The system consists of two sets of plasmids, an effector module and a reporter module, both sets assembled with regulatory components (as promoter and terminator regions) appropriate for expression in mammals, including humans, and plants. The complete method involves plasmid design, DNA delivery, testing and both molecular and phenotypical assessment of results. This platform presents a suitable workflow for the identification and functional validation of new tools for the genetic regulation and reprogramming of organisms with importance in different fields, from medical applications to crop enhancement, as shown by the initial results obtained. This protocol can be completed in 4 weeks for mammalian cells or up to 8 weeks for plant cells, considering cell culture or plant growth time.

## Introduction

The ability to regulate gene expression in response to external cues is one of the central mechanisms of the differentiation and maintenance of life in nature, as well as one of the main goals of scientists in efforts to control and reprogram organisms. Therefore, the availability of molecular tools that allow genetic manipulation is crucial for advances in synthetic biology, especially in creating intricate genetic circuits and activation cascades to work as synthetic regulatory networks. A prominent group of effectors used to that end are integrases, a superfamily of site-specific recombinases (SSRs) capable of directed and controlled rearrangement of DNA sequences [1–4]. Although tyrosine integrases such as Cre/LoxP [5–8] and λ Int [5] systems have historically been predominantly used, recently, another family of SSRs known as serine integrases has received attention, in great part because of the advantages they present over their counterparts. The main advantages for application in synthetic biology include the shorter length of their attachment sites, unidirectional recombination and nondependence on auxiliary effectors to work [9–14]. Despite the advantages, only a very limited number of functional serine integrases have been available for use in eukaryotic organisms. We have recently used 6 out of the 13 newly described functional serine integrases previously identified and characterized in *Escherichia coli* by Yang and collaborators [15] to assemble and test genetic switches capable of modulating gene expression upon Int activation in eukaryotic systems, including plant-, bovine- and human-derived cells [16]. While in the original work in *E*. *coli* all integrases showed similar efficiency, their efficiency in plant and mammal cells varied considerably, yet successful recombination was detected for all integrases used.

**Fig 1:**
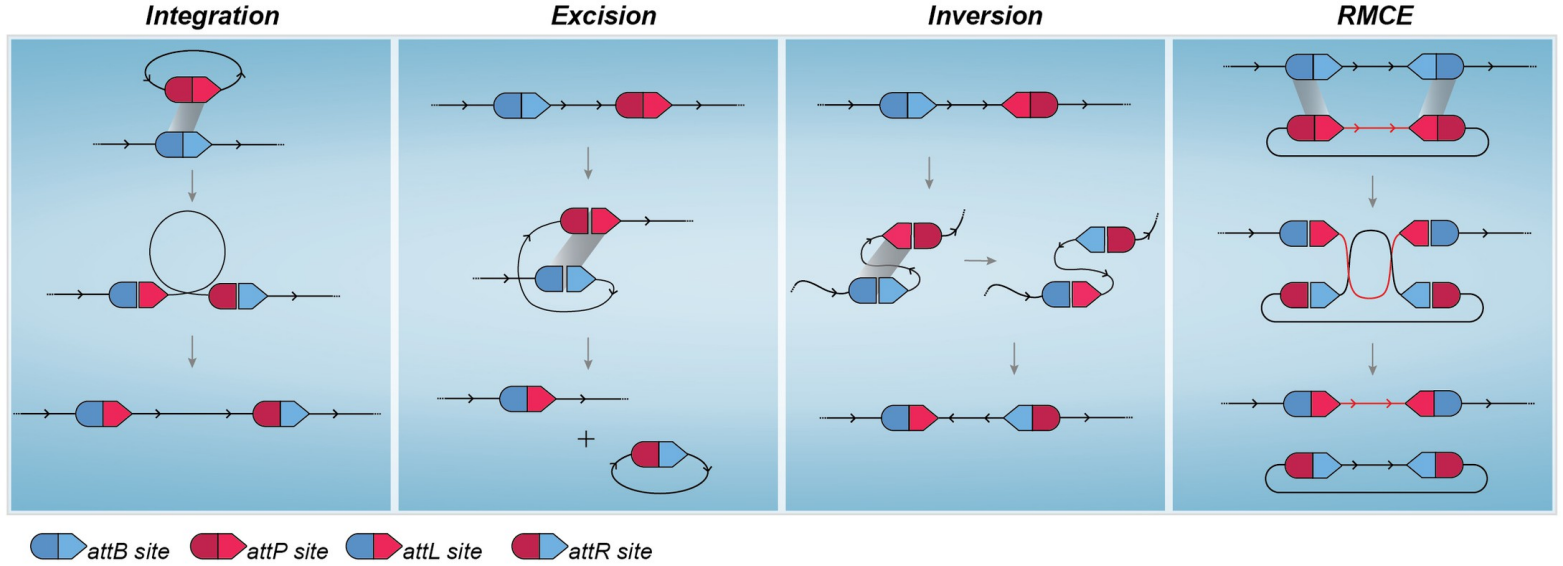
Serine integrase mediated DNA rearrangement. (A) Although the original function of this class of recombinases in nature is the integration of bacteriophage DNA into a host genome upon recombination of *attB* and *attP* sites, synthetic manipulation of *att* sites can lead to different outcomes. Recombination of *att* sites located in the same molecule will result in (B) Excision of flanked DNA region if both sites have the same orientation or (C) Inversion when sites are present in opposite orientations. (D) Recombinase Mediated Cassette Exchange (RMCE) allows the exchange of a large sequence of DNA when a molecule harbors two copies of an *attP* site and a second molecule harbors two copies of the cognate *attB* site.

Originally present in nature as a mechanism used by some bacteriophages to integrate their DNA into the genome of a prokaryotic host, the serine-integrase mechanism of action involves recognition and physical interaction with a specific pair of attachment sites present in both phage and bacterial host DNA known as *attP* and *attB* sites, respectively [17, 18]. Upon binding to DNA, conformational alterations of the complex help to expose the central part of the *att* sites, known as the core sequence, to the catalytic site responsible for the double break in the DNA strands. Subsequent conformational changes lead to the recombination of cut ends from complementary *att* sites and final ligation to form the newly recombined *attL* and *attR* sites in a permanent, unidirectional way [19–21]. Since this whole process occurs without the need for any additional endogenous effector, serine integrases exhibit valuable host plasticity when considered as a gene editing tool. However, it is the rational manipulation of *att* site combination and orientation that truly broadens the use possibilities of these recombinases. When cognate *att* sites are present in different DNA molecules, the natural integration process occurs. Notwithstanding, if *att* sites are synthetically designed to be present at the same molecule, the recombination of these sites can have varying outcomes according to their orientation, namely, excision of a portion of the DNA molecule, a 180° inversion of a DNA sequence flanked by the *att* sites [15] and recombinase-mediated cassette exchange (RMCE) [22, 23]. Diagrams to better illustrate these possible outcomes are presented in Fig 1.

Genetic switches designed to invert a given sequence are especially interesting for expression control. In this type of system, a gene of interest or the promoter regulating its expression is assembled in an opposing orientation relative to the other and flanked by a pair of *att* sites. Upon integrase activation, *att* sites are recombined with consequent inversion of the DNA sequence in between, therefore allowing proper gene transcription [15, 16]. Recombination by serine-integrase is unidirectional and permanent unless a cognate recombination directionality factor (RDF; an accessory protein encoded in the same phage genome that contains the one integrase with which it can interact) [24] or bidirectional activating mutations [25] are present.

Although the most prominent applications of recombinases to date involve the use of tyrosine-integrases, serine-integrases were first identified and used to deliver DNA in *Streptomyces spp*. over 30 years ago [26]. Since then, many works have taken advantage of their recombination capabilities in prokaryotic models [15], unicellular eukaryotic organisms [23], and in a few cases, complex multicellular organisms, including mouse and human cells [16, 27], and they have even been used as molecular tools for in vitro DNA plasmid assembly [14, 28]. More recently, the serine-integrase ability to excise or flip terminators and genes has been more widely applied in the assembly of genetic circuits designed to either modulate gene expression or compute events by permanently rearranging DNA parts based on binary logic [15, 29–32]. The higher intricacy of activation cascades and genetic circuits with multiple integrases as effectors intensifies the demand for new functional integrases and corresponding *att* sites, encouraging efforts to describe new members and to develop systematic models for the identification and testing of candidates, such as bioinformatic tools to identify potential serine-integrases and *att* sites in genome databases [15, 27] and *in silico* platforms to help with the design and assembly of complex genetic circuits [33–35]. That need is even more accentuated when considering the use of genetic circuits based on integrases in eukaryotic organisms, since the higher complexity and compartmentalization create a functional bottleneck in which many integrases that function in bacteria do not work or present cytotoxic effects in eukaryotic systems [16, 23]. Xu et al. [36] showed that some integrases able to rearrange sequences on plasmid DNA in human cells lost their ability to do so when the target DNA was integrated into the genome, while Andreas et al. [37] showed that alterations such as adding a nuclear localization signal (NLS) at the ΦC31 C-terminus region enhanced its editing capabilities in mouse cells. As mentioned, we also showed that integrases with similar efficiency levels in bacteria showed varying degrees of efficiency when tested in plant and animal cells. Moreover, despite the increasing levels of interest and publications using serine-integrases in eukaryotic organisms, very few new members have been validated in these systems, with many groups always working with the same limited pool of candidates, mainly φC31, BxB1, and less frequently, φR4 and TP901. Considering this scenario, we propose in this protocol a platform to systematically test and identify functional serine integrases for the assembly of genetic circuits in different plant and mammalian organisms.

**Fig 2:**
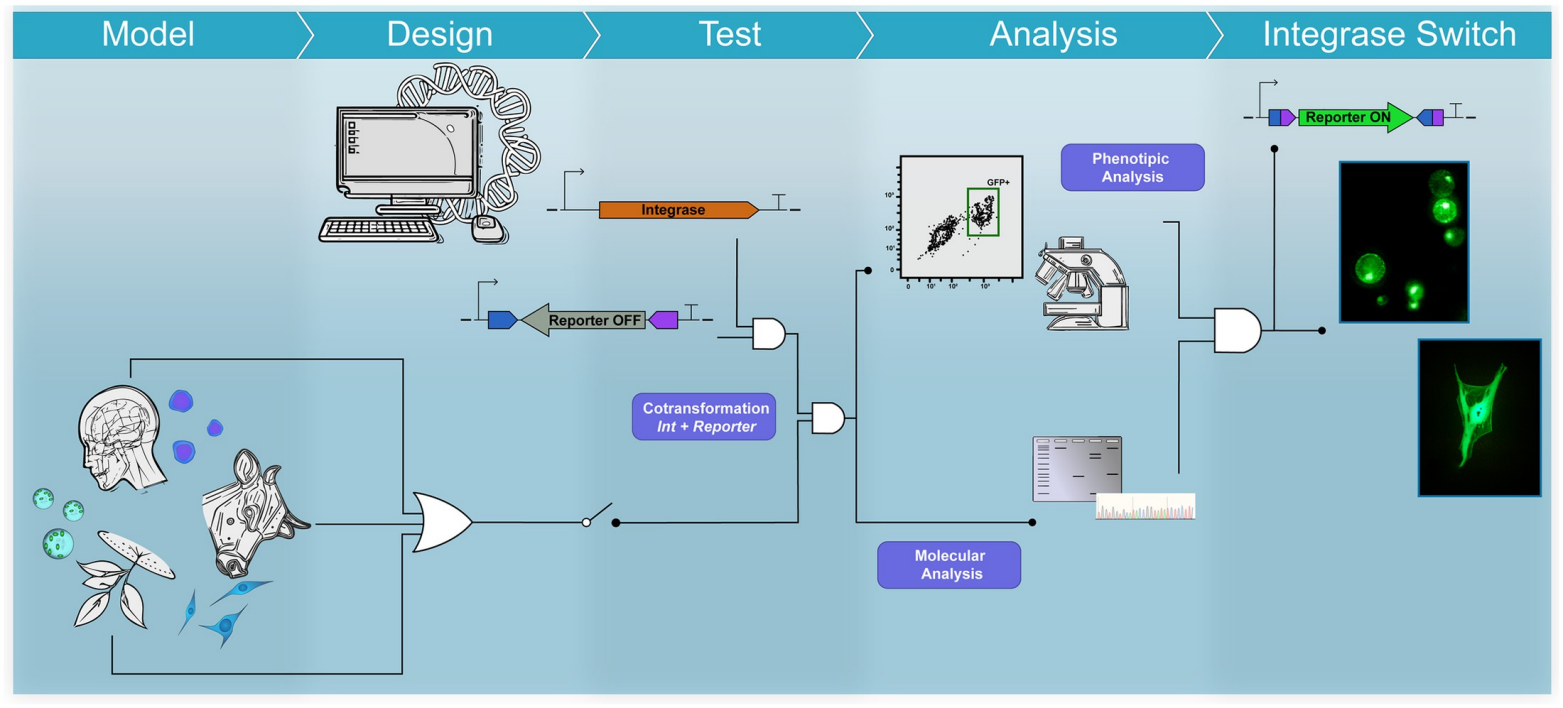
Strategic overview of the serine integrase-based platform for the functional characterization of genetic switch controllers in eukaryotic cells.

The platform can be seen as a workflow comprising six main stages: I) *in silico* design and synthesis of plasmid constituents of each effector and reporter module; II) cell acquisition and culture maintenance, which can be subdivided into in vitro culture of established mammalian cell lines, patient cell isolation and plant growth for protoplast isolation according to the model selected; III) DNA delivery; IV) cytotoxicity evaluation; V) molecular analysis, including DNA preparation, primer design, PCR setup and sequencing; and VI) phenotypic analyses by fluorescence microscopy and flow cytometry. Fig 2 shows a schematic overview of the workflow proposed.

## Materials and methods

The protocol described in this peer-reviewed article is published on protocols.io [dx.doi.org/10.17504/protocols.io.rm7vzx945gx1/v1] and is included for printing purposes as S1 File. Subprotocols specific for each organism group used in the present work are included for printing purposes as S2–S5 Files.

### Ethical statement

All work involving cells derived from mammalian organisms must comply with and be performed under strict ethical guidelines. Experiments with human materials must conform to all relevant institutional and governmental ethics regulations, and appropriate informed consent must be obtained for the use of human blood or patient-derived materials. For the development of the present protocol, Peripheral Blood Mononuclear Cells from healthy donors were collected and used upon approval by the Brazilian National Cancer Institute (INCA) Ethics Committee and signing of Review board-approved informed consent forms by the donors. Experiments involving the use of human-derived stem cells were approved by the ethics committee of Copa D’Or Hospital (CAAE number 60944916.5.0000.5249, approval number 1.791.182). Regarding the acquisition and use of bovine primary fibroblasts, all experimentation performed was approved by the Ethics Committee on the Use of Animals (CEUA) of Embrapa Genetic Resources and Biotechnology (Brasilia, Brazil) in March 2013 (approval reference no. 001/2013).

### Experimental design

#### *In silico* design of plasmids

**Fig 3:**
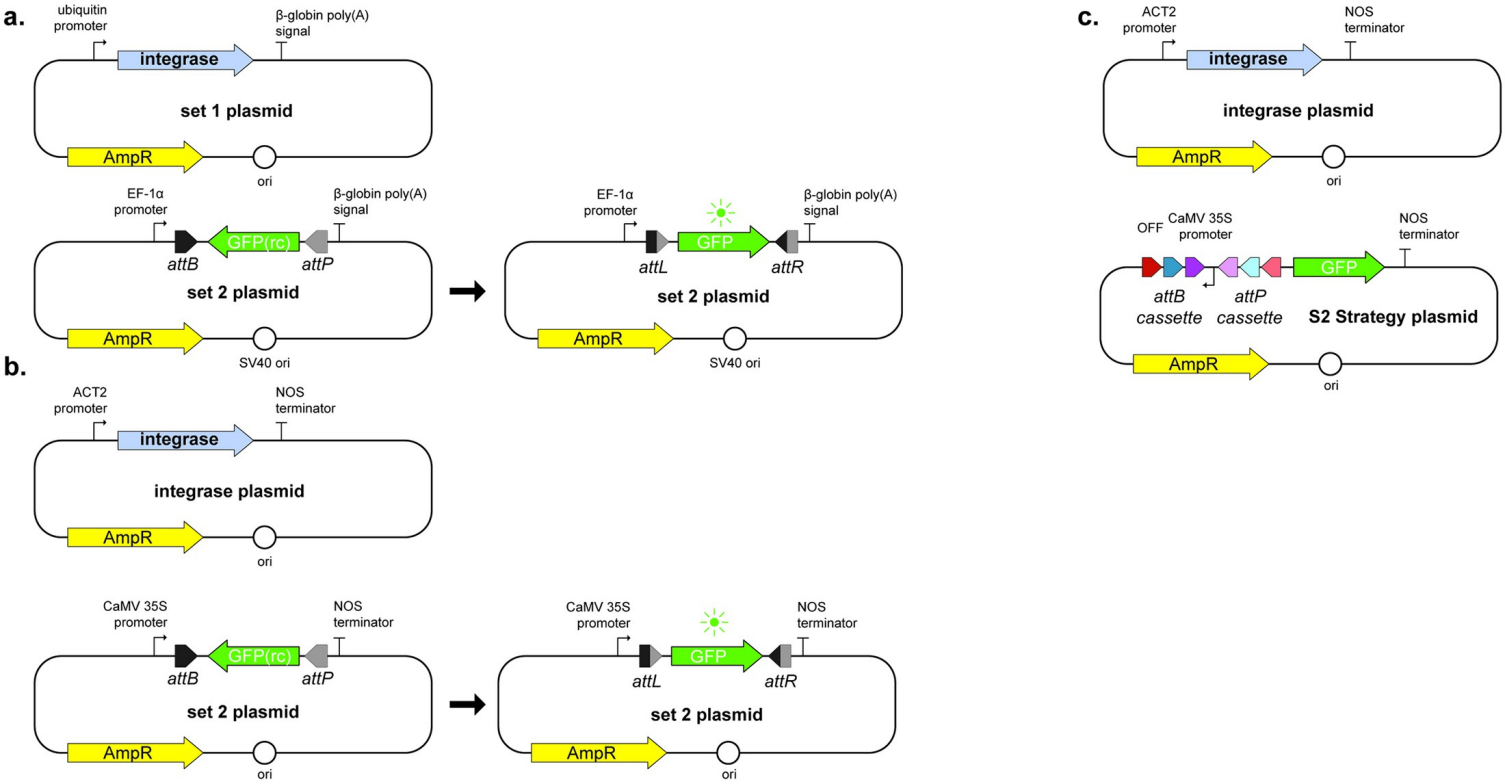
Designed unidirectional genetic switches composed of two sets of synthesized plasmids. (a) and (b) Plasmid sets for S1 strategy in mammalian and plant models, respectively. (c) Plasmid sets for S2 strategy applied for plant models. Here, the promoter sequence, not the *gfp* gene, is under the control of integrase-mediated inversion and activation. Figure adaptation from Gomide et. al., 2020 [16].

These initial steps are crucial for a successful experiment since they include the definition of all the regulatory parts to be used, as well as the rational design of the switch to be tested. The modular aspect of the construction allows the easy and fast assembly of numerous plasmid sets carrying different integrases and their respective reporter cassettes. As indicated in the schematics presented in Fig 3, the basic expression unit for the effector plasmids consists of the promoter sequence most appropriate to the organism in which the test will be performed, followed by the integrase gene and a terminator sequence downstream. For better expression rates, the integrase gene sequence may be codon optimized before synthesis to match the codon usage patterns of the organism or group of interest. This expression unit must be inserted into an appropriate backbone that allows cloning and transformant selection for the preparation of DNA in bacteria before assays and proper behavior once delivered into the eukaryotic model. The promoter sequence must render constitutive expression, and weak promoters, i.e. sequences known to promote low transcription rates- therefore affecting mRNA accumulation-, are avoided to reduce the chance of falsely identifying an integrase as nonfunctional in the system when in reality it is due to low expression levels. Additional elements, like RBS and enhancer sequences can be screened and used to fine-tune transcription and increase integrase expression In this protocol, we selected the promoters pUbC [38] and pAct [39] for use with mammalian and plant cells, respectively.

The reporter unit has a more complex design but still follows the modular aspect of the effector plasmids with the classic downstream sequence of promoter, gene of interest and terminator sequence. The promoter and terminator sequences selected for this protocol in the reporter module were pEF1a [38] and B-globin poly(A) signal for use with mammalian cells and pCaMV35S and NOS terminator for use with plant cells. *gfp* was used as the reporter gene here, although the choice of reporter must take into consideration background fluorescence wavelength and possible emissions from cell constituents such as chloroplasts in the plant cells to avoid emission overlap. Two reporter strategies were designed and named S1 and S2 Strategy. S1 strategy (Fig 3A and 3B) is designed to assess the integrase’s capacity for rearranging a coding DNA sequence orientation, for which the reporter gene sequence must be inserted into the construct in an anti-sense position in relation to the promoter, thus in a silenced state—here referred to as the OFF STATE—and flanked by the integrase recombination sites. It is crucial that recombination sites are inserted in opposite directions to each other to ensure inversion of the DNA sequence between them given the recombination dynamics discussed above. The selection of integrases and their cognate *att* site pair should involve considering the eventual presence of starting codons in the *att* sites or recombined *attL* and *attR* site sequences that can interfere with gene expression and reading frames. In the occurrence of such codons, swapping *attB* and *attP* positions can be considered, as long as the final design contains the most downstream site in its reverse complement form.

The genetic switch can be assembled to direct the reorientation of genetic parts other than a gene of interest. In the S2 strategy designed for this platform, the promoter is initially inactive due to its opposite orientation in relation to the reporter gene (Fig 3C). Another improvement in this strategy is the use of a cassette of *att* sites in tandem, which allows the use of the same reporter plasmid to test the activity of different integrases or sequential induction events with different integrases to invert the flanked sequence back to its initial position. Despite broadening the applications of the genetic switch, this strategy demands prior knowledge of the integrases selected or a more thorough evaluation and proper use of controls to ensure their orthogonality.

All plasmids used in the development of this protocol are listed in Table 1 below, with Addgene database accession number for more information including annotated sequence maps. The genetic parts in the constructions can be changed to better suit different organisms.

**Table 1:** Plasmids used in the assembly of S1 and S2 strategy sets.

Plasmid Set	Name	Addgene ID
pSG	INCTbiosyn-pEF-gfp(rc)2	127504
	INCTbiosyn-pEF-gfp(rc)4	127505
	INCTbiosyn-pEF-gfp(rc)5	127506
	INCTbiosyn-pEF-gfp(rc)7	127507
	INCTbiosyn-pEF-gfp(rc)9	127508
	INCTbiosyn-pEF-gfp(rc)13	127509
	INCTbiosyn-pEF-gfp(rc)phiC31	127510
	INCTbiosyn-pEF-gfp(rc)Bxb1	127511
pIE	INCTbiosyn-pUB-HspINT2	127512
	INCTbiosyn-pUB-HspINT4	127513
	INCTbiosyn-pUB-HspINT5	127514
	INCTbiosyn-pUB-HspINT7	127515
	INCTbiosyn-pUB-HspINT9	127516
	INCTbiosyn-pUB-HspINT13	127517
	INCTbiosyn-pUB-HspINTphiC31	127518
	INCTbiosyn-pUB-HspINTBxb1	127519
pSP	INCTbiosyn-p35S(rc)2_4_5-gfp	127520
pSG	INCTbiosyn-p35S-gfp(rc)2	127521
	INCTbiosyn-p35S-gfp(rc)4	127522
	INCTbiosyn-p35S-gfp(rc)5	127523
	INCTbiosyn-p35S-gfp(rc)7	127524
	INCTbiosyn-p35S-gfp(rc)9	127525
	INCTbiosyn-p35S-gfp(rc)13	127526
	INCTbiosyn-p35S-gfp(rc)phiC31	127527
	INCTbiosyn-p35S-gfp(rc)Bxb1	127528
pIE	INCTbiosyn-pAct-AtINT2	127529
	INCTbiosyn-pAct-AtINT4	127530
	INCTbiosyn-pAct-AtINT5	127531
	INCTbiosyn-pAct-AtINT7	127532
	INCTbiosyn-pAct-AtINT9	127533
	INCTbiosyn-pAct-AtINT13	127534
	INCTbiosyn-pAct-AtINTphiC31	127535
	INCTbiosyn-pAct-AtINTBxb1	127536

Cell acquisition and culture maintenance. Another important aspect of the presented protocol is the plasticity in regard to eukaryotic cell models with which it can be performed. Primary bovine fibroblasts were obtained from 14-month-old *Bos indicus* bull oxtail by biopsies following the protocol defined by Freshney [40] with modifications. Briefly, recovered pieces of tissue are washed with 0.05% trypsin to detach cells, which are then cultured in Dulbecco’s Modified Eagle Medium supplemented with fetal bovine serum 10% at 37°C and 5% CO2 atmosphere in culture flasks for at least three passages or until a homogeneous culture of attached fibroblasts can be observed. HEK293T cells can be purchased from cell line culture collections and stored, although some culture passages are recommended prior to cell transfection to ensure that metabolically fit cultures are being used. For human donor primary or derived cells used to pursue potential medical applications of the genetic switches, PBMCs were isolated from donor blood via Ficoll density gradient centrifugation, while neural stem cells and human embryonic stem cells from the BR-1 cell line were cultured in neural induction medium (NEM, Advanced DMEM/F12 and neurobasal medium (1:1) with neural induction supplement). All animal and human donor-derived cells and experimentation performed with them must undergo appropriate ethics committee evaluation and approval.

Plant cells derived from *A*. *thaliana*. Briefly, protoplasts were isolated from young leaves of 5-week-old plants cultivated at 22°C under a 12 h light/12 h dark regimen according to the protocol described by Yoo et al. [41] with modifications. The collected leaves were prepared by making shallow cuts on the adaxial face with a scalpel to enhance internal exposure to the enzymatic solution. In this work, *A*. *thaliana* leaf cells were disaggregated using an enzymatic cocktail of 0.2% pectolyase, 0.5% driselase and 1.5% cellulase, but other combinations and adaptations are possible to better digest the cellular walls of cells from other species. The age and overall morphology and health of leaves can have a great impact on protoplast isolation; therefore, the use of young specimens of up to 60 days is recommended.

#### DNA delivery and controls

Given the particularities of each model, different plasmid delivery strategies are applied depending on the cell line. In general, nonintegrative transfection resulting in transient expression of the effector integrase and reporter gene is sufficient for the functional screening proposed in this protocol. All strategies applied here have in common the concurrent transfection of effector and corresponding reporter plasmids in equimolar ratio and a period of at least 24 h post-transfection before assessing the outcome responses. Different delivery strategies were used depending on the model. Transfection of HEK293T cells and primary bovine fibroblasts was performed by lipid-mediated delivery using Lipofectamine LTX and Plus reagent (Invitrogen) according to the manufacturer’s recommendations. Despite being widely used and considered a gold standard method for DNA delivery [42], lipofectamine transformation can result in low transformation efficiency and high cytotoxicity for some cell lines, including human primary cells and non-adherent cultures. Hence, PBMC, NCS and hES cell transformation was carried out by electroporation using an optimized adaptation of the nucleofection method established by Chicaybam et al. [43]. This method yields higher transformation efficiency for some human cells, including primary and stem cells, with low cytotoxic effects at a lower price than the classical nucleoporation method. For plant protoplasts, CaCl2-PEG chemical transfection [44, 45] was used to carefully deliver the plasmid sets. Although other methods exist for DNA delivery in protoplasts, including agrobacteria-mediated transformation [46] and electroporation [47], PEG-mediated transfection is the most used delivery system due to its easy manipulation, low cost and no need for specific equipment. Another important advantage of this method is that it allows for more gentle manipulation of cells, an important aspect when handling fragile protoplasts following cell wall digestion.

Although the cotransformation of an effector and corresponding reporter plasmid pair is the central aspect of the functional evaluation of an integrase, many control groups containing different plasmid combinations are necessary to validate the obtained results. The first set of controls will be the samples to which only one member from the plasmid pair is delivered to eliminate any background signal or endogenous machinery activity interference that could lead to false-positive results. With these samples, we will have the opportunity to assess fluorescence levels coming from a non-activated reporter in the absence of the effector integrase, for instance, which could be indicative of a leaky system or unwanted promoter activity from the downstream *att* site present. One important example of such occurrence is the observed promoter activity of Int13 *attP* site described by Yang et al. in *E*. *coli* [15]. An unlikely, although not impossible, fluorescence emission by the integrase itself could also be ruled out in this context. These control groups are also important to identify potential cytotoxic effects from any of the components of the system, especially from the integrase being tested, which may cause DNA damage or integration at endogenous pseudosites [36, 48, 49], disrupting the proper expression of important unrelated genes. It is important to note that for these controls, the plasmid absent from the original pair must be replaced by the same amount of a mock plasmid, normally the empty backbone used in the constructions, to maintain the DNA load concentration and molar ratio. Another set of controls is especially necessary in the case of our S2 strategy or when future applications of the integrases being tested involve the simultaneous presence of more than one in a genetic switch or cascade: in this case, combinations of an effector plasmid with one integrase should be cotransformed with the reporter plasmid carrying recombination sites of a different integrase to assess orthogonality, i.e., one integrase is not capable of interacting with or rearranging *att* sites other than their cognate site pair.

Positive controls included plasmids carrying unaltered reporter gene sequences under constitutive expression and the design and synthesis of the expected rearranged cassette carrying the reporter gene sequence in its forward orientation flanked by the expected recombined *attL* and *attR* sites. The first group is a classic positive control to ensure cell behaviour and allow equipment calibration. As for the constructions with *attL* and *attR* sites, it is an important group to help evaluate if an observed lowered level or lack of signal from the reporter following integrase activation indicates a negative result or inhibitory transcriptional interference due to the presence of the post-recombination sites. Although serine-integrases are not able to recombine *attL*/R sites, Chao et al. demonstrated that integrase interaction with these sites can alter target expression [50].

As mentioned, the use of the first set of control groups is suitable for evaluating cytotoxicity. Given that one of the steps in the recombination of *att* sites by an integrase involves double-strand breaks in the DNA, the occurrence of off-target activity at pseud-sites causing unintended DNA damage can be a concerning source of toxicity and limit the use of such integrase in the assembly of a genetic switch. Cell integrity markers and colorimetric assays designed to measure cell viability are an easy way to check for deleterious effects. PBMCs and stem cells used were stained with 7-AAD, a live cell impermeable fluorophore that allows the quantification of stained dead cells in the same flow cytometry run used to assess reporter expression. A similar strategy was applied to protoplasts by staining cells with fluorescein diacetate (FDA), although in this case, live cells were stained. For HEK293T cells and primary bovine fibroblasts, an enzymatic MTT assay was performed.

### Molecular analyses

**Fig 4:**
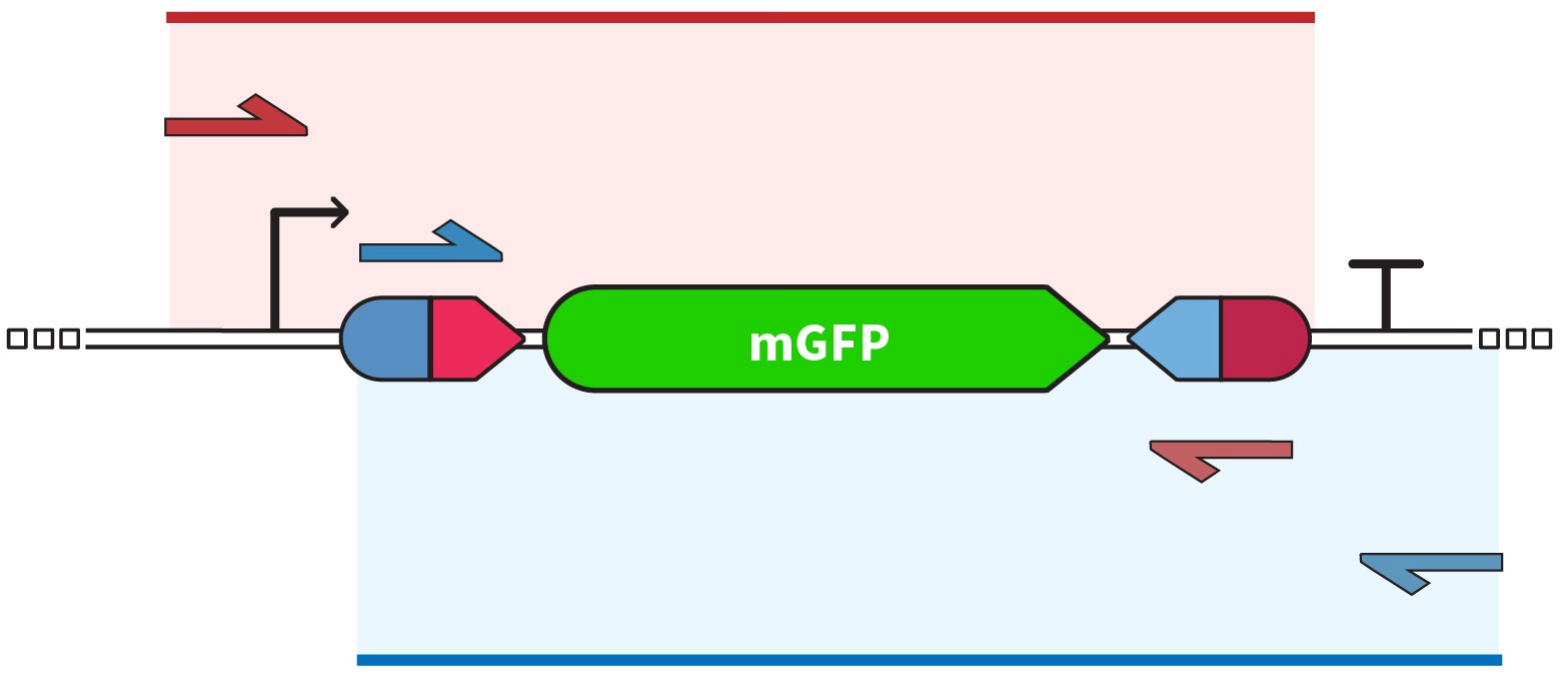
Oligonucleotide pairs and expected amplification regions in the two-amplicon strategy. Figure adaptation from Gomide et. al., 2020 [16].

To confirm integrase activity, both molecular and phenotypical analysis methodologies should be applied. As we observed previously [16], when screening for integrases capable of rearranging DNA in eukaryotic cells, sometimes the confirmation of sequence inversion by amplification and sequencing was not accompanied by a positive reporter signal. DNA extraction and amplification should follow standard practices established for Sanger sequencing, but primer design must be carefully performed. In addition to confirming DNA inversion, amplification and sequencing are means to confirm proper recombination of *att* sites by directed editing of the integrase not causing point mutations or DNA damage at the core sequencing, gene of interest or surrounding regions. A good strategy is to amplify the 5’ end of the gene and upstream formed *attL* site as one fragment and the 3’ end of the gene and the downstream *attR* formed site as another fragment, keeping an overlap region between them to ensure good coverage of the whole cassette, as shown by the diagram in Fig 4. It is important to note that when opting to use primers annealing to post recombination *attL* and *attR* sites to increase specificity, the primers must have a high Tm point and PCR setup performed with higher annealing temperatures since part of the primer will inevitably anneal to respective *attB* and *attP* sites to a certain extent.

Obtained amplicons must be sequenced to ensure amplification specificity and correct formation of *attL* and *attR* sites with target coding sequence inversion. For most standard model organisms, especially cell cultures with high transformation efficiency, amplification should be no problem and Sanger sequencing will provide good results whilst still easily accessible in facilities or sequencing companies at low costs. A recommendation is to have fragments sequenced with forward and reverse primers to allow better coverage of both ends of the region of interest. Overlap of these sequences can also help identify mutations from sequencing error instead of integrase-cause DNA damage. However, sequencing method selection can be affected by other factors. When working with cells of low transformation efficiency that could impair DNA delivery, tissues instead of cell culture that can result in mixed population with more non-transformed than transformed cells, or screening integrases with very low recombination efficiency, a more robust and sensitive sequencing method may be used. Nanopore DNA sequencing is a good alternative in these scenarios [51], more easily detecting low copy numbers of edited sequences in a mixed population. This methodology present other advantages when compared to Sanger sequencing, including the possibility to better evaluate the ocurrence and rate of integrase-related mutations and DNA damage due to its high accuracy and possibility of reads quantification for integrated stoichiometry analysis.

### Phenotypical analyses

Reporter activation can be assessed by fluorescence visualization or quantification. Fluorescence microscopy is a fast way to observe reporter expression activation but will result only in qualitative data. For accurate quantification to allow efficiency ranking between all integrases tested flow cytometry must be performed. Multiple time points should be evaluated to identify the best incubation period for each model. For the models used in our work, 48h was defined as standard incubation time, with eGFP signal decrease after 72h post-incubation in mammalian cells. Due to the short survival in culture conditions, flow cytometry of treated protoplasts was performed after 24 hours following transformation. High event count must be used, and all control groups must be considered to ensure a proper definition of gates to differentiate between eGFP positive and negative cell populations.

Assays must be done with at least three independent biological replicates; technical replicates should be included to account for measurement and equipment variations. Statistical method choice must be done considering the specificities of each new model and experimental design selected. For our platform, quantitative data were analyzed using the nonparametric Kruskal—Wallis test, with Dunn’s test as post hoc for pairwise comparisons. Although One-way ANOVA is typically used for multiple comparisons, nonparametric tests do not rely on assumptions regarding normal distribution and variance homogeneity, which can be hard to confirm when working with small sample sets.

### Expected results

We first developed our platform to assess the functionality of 6 integrases previously characterized only in *E*. *coli*, named Int2, Int4, Int5, Int7, Int9 and Int13 [15]. Original bacterial host and sequence of *attB/attP* sites for each Int are presented at Table 2. However, the plasticity of our workflow makes it suitable for the initial screening in eukaryotic cells of any other new serine integrases identified in silico from genome databases or with ones previously tested only in prokaryotic cells with very little adaptation needed.

**Table 2:** Bacterial host and sequence of attachment sites for the six integrases used by Gomide and collaborators (2020) [16].

Integrase	Host	attB (5’– 3’)	attP (5’– 3’)
Int2	*Streptomyces scabiei* 87.22	ggacggcgcagaaggggagtagctcttcgccggaccgtcgacatactgctcagctcgtc	gctcatgtatgtgtctacgcgagattctcgcccgagaacttctgcaaggcactgctcttggct
Int4	*Streptococcus equi subsp equi* 4047	ttccaaagagcgcccaacgcgacctgaaatttgaataagactgctgcttgtgtaaaggcgatgatt	caaaaattacaaagttttcaacccttgatttgaattagcggtcaaataatttgtaattcgttt
Int5	*Streptomyces phage* PhiK38-1	gagcgccggatcagggagtggacggcctgggagcgctacacgctgtggctgcggtcggtgc	ccctaatacgcaagtcgataactctcctgggagcgttgacaacttgcgcaccctgatctg
Int7	*Geobacillus sp* Y412MC61	agacgagaaacgttccgtccgtctgggtcagttgggcaaagttgatgaccgggtcgtccgttcctt	ggtgttataaacctgtgtgagagttaagtttacatgcctaaccttaacttttacgcaggttcagctt
Int9	*Staphylococcus aureus* str. Newman	tttatattgcgaaaaataattggcgaacgaggtaactggatacctcatccgccaattaaaatttg	gtggttgtttttgttggaagtgtgtatcaggtatctgcatagttattccgaacttccaatta
Int13	*Bacillus cytotoxicus* NVH 391–98	cgcatacattgttgttgtttttccagatccagttggtcctgtaaatataagcaatccatgtgagt	caataacggttgtatttgtagaacttgaccagttgttttagtaacataaatacaactccgaata

Adaptation from Yang et al (2014) [15].

**Fig 5:**
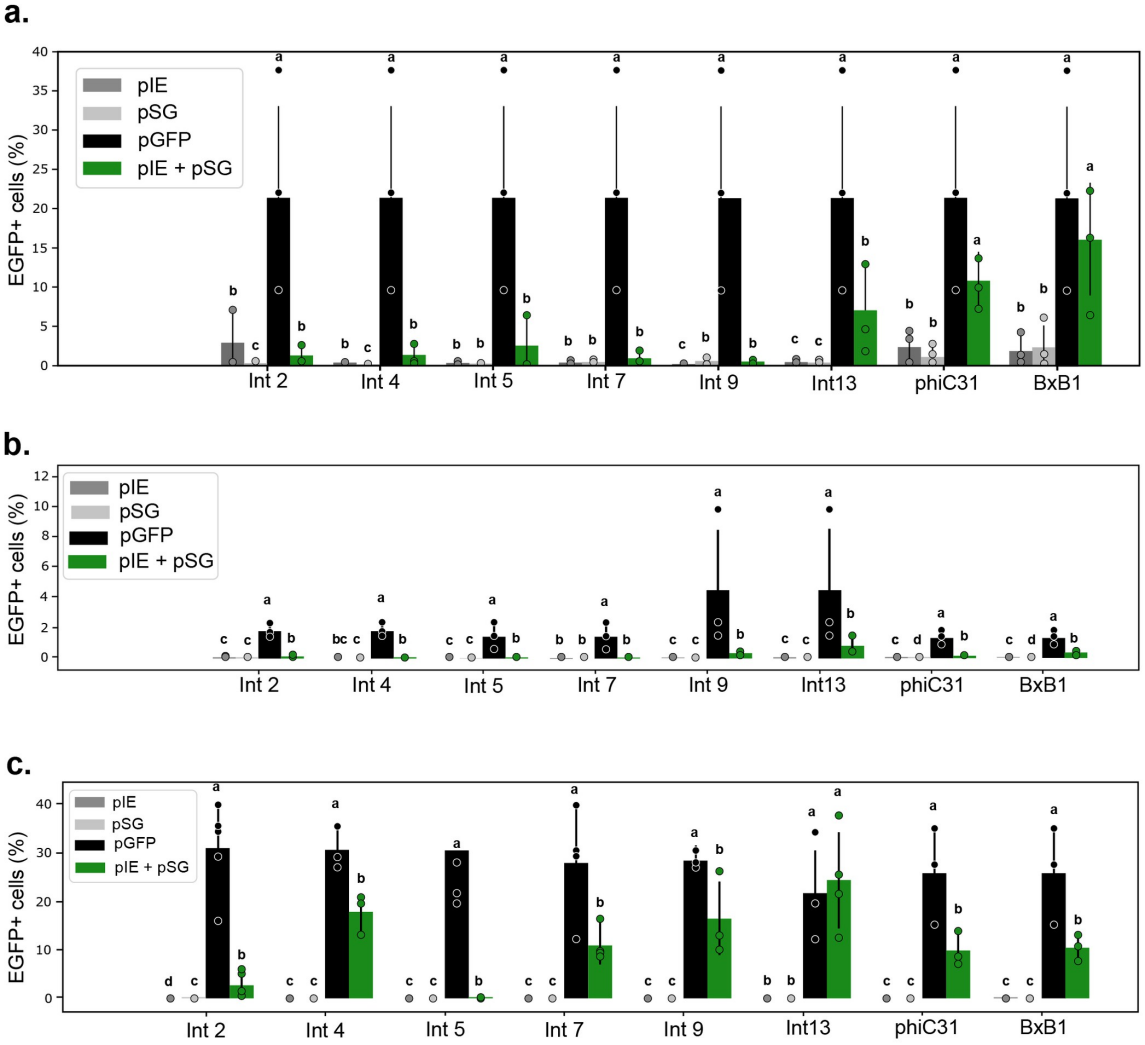
EGFP fluorescent cell ratio between Int S1 treatments in the different eukaryotic systems analyzed (a, human cell; b, bovine fibroblasts cells; and c, *A*. *thaliana* protoplasts). pIE: effector Integrase Expression plasmid; pSG: reporter Switch GFP plasmid; pGFP: positive control with constitutive expression of *gfp*. Figure adaptation from Gomide et. al., 2020 [16].

In our work [16], we were able to confirm the successful recombination of recognition sites and formation of correct *attL* and *attR* sites with consequent inversion of the *egfp* gene used as a reporter for all the integrases studied. Despite the positive results at the molecular level, *mgfp* expression levels after switch activation varied depending on the integrase and organism used when cells were analyzed by flow cytometry or fluorescence microscopy. Overall, for the S1 experimental design Int13 switches resulted in higher levels of fluorescence emission for all the organisms used, followed by Int9 and Int4 in bovine fibroblasts and protoplasts. Int5 switch yielded the lowest levels of signal despite molecular confirmation of DNA inversion, possibly an indication of low frequencies of recombination in the systems (Fig 5). Integrases phiC31 and BxB1, used for comparison given their well established applications in mammal and plant systems, also resulted in varying signal levels depending on the cell used, although overall results were similar to the ones obtained when using Int13. Interestingly, the S2 promoter switch construction led to a result compatible with the S1 GFP switch in plant protoplasts. However, Int2 exhibited a much higher number of EGFP-positive cells when the promoter was flipped than when the targeted gene was [16].

These analyses have proven the efficiency of our Int-based platform for the functional characterization of these enzymes, as well as its robustness for the further investigation of Ints as genetic switch controllers in eukaryotic cells. Considering that all six integrases showed similar efficiency compared to each other when first used in *E*. *coli*, the broader range of efficiency obtained in eukaryotic cells can be advantageous when considering the assembly of multicomponent circuits, where variations on potency can help regulate flow rates at defined points in a cascade, allowing a fine-tuning of the system.

Although not further investigated in the present work, the variations in *gfp* expression levels observed both amongst integrases or between mammalian and plant models were partially unexpected. Some works studying integrase applications on eukaryotic models concluded that such variations could occur not only between distant groups like mammalian cells and plants or yeast but also when comparing more closely related organisms, as reported by Xu et al. (2013) [36] when comparing the use of more than ten Ints in human and murine cells. One example can be easily observed with Int TP901: In the mentioned work, this Int was found functional in human cells but incapable of rearranging DNA in mouse stem cells. The same group reported its functionality in Saccharomyces cerevisiae with considerably higher efficiency [23], while Guiziou et al. (2023) [52] found no TP901 activity in Arabidopsis. Despite reporting and discussing these variations, none of the works presented a deeper investigation into their biological causes. Possible clues can be found in the work published by Chao and collaborators (2021) [50]. They measured recombination kinetic parameters for six Ints, including TP901, to propose a prediction model for systematically reporting Int recombination activity. One important finding in their work is that different Ints can present varying expression levels under the same promoter, and Int concentration can also impact the recombination rate. For instance, phiC31 showed higher recombination rates when under transcriptional control of a weaker Ub promoter than when controlled by the strong CMV promoter. These results highlight the complexity of Int efficiency control and how organism-dependent molecular context can affect Int behavior.

### Limitations

Although the Int-based platform proved efficient and robust for the functional validation of integrases in eukaryotic cells, some limitations exist. Studies have revealed that Ints are typically very specific to their cognate *att* sites. However, Int shows low levels of off-target activity at pseudo-site sequences similar to their natural *att* sites [17, 53–55]. Additionally, Ints can cause residual DNA damage or mutations when acting in cells that are not their natural hosts. Various types of damage have been found, including mutations and deletions of the *att* sites, which can make them refractory to later reactions [56]. Cell cytotoxicity is another limiting factor observed when using Ints [23, 36].

As for technical limitations in our platform, the main one we should consider is the discovery of new Ints to be tested since a complete system involves not only the integrase itself but also the identification of the cognate *attB*/*attP* site pair, which can be more complex to identify than the recombinase gene [15]. The use of RDFs to allow controlled bidirectional recombination is another factor that can broaden Int applications and should be considered. However, limitations including the need of one specific RDF protein for each new serine-integrase, the availability of only a few Int-RDF pairs identified at the moment and laborious validation and optimization required to establish new tools present significant challenges for their use. Regarding analytics, implementing quantitative methods for molecular detection of DNA rearrangement like qPCR assays can bring another valuable parameter for Int efficiency classification. The use of a more robust method for sequencing, like nanopore NGS, can be an alternative in some situations, especially when dealing with low recombination efficiency or a mixed population of edited and not edited targets if an inadequate representation of edited molecules could be preventing identification of DNA rearrangement.

## Supporting information

S1 File Step-by-step protocol collection. (PDF)

S2 File Step-by-step protocol for assembly of a serine integrase-based platform for functional validation of genetic switch controllers in eukaryotic cells-human. (PDF)

S3 File Step-by-step protocol for assembly of a serine integrase-based platform for functional validation of genetic switch controllers in eukaryotic cells-animal. (PDF)

S4 File Step-by-step protocol for assembly of a serine integrase-based platform for functional validation of genetic switch controllers in eukaryotic cells-plant. (PDF)

S5 File Step-by-step protocol molecular analyses. (PDF)
