# Supplementary material for: Protocol for the establishment of a serine integrase-based platform for functional validation of genetic switch controllers in eukaryotic cells
Source: PLoS One. 2024 May 23;19(5):e0303999. doi: 10.1371/journal.pone.0303999 (PMC11115199; doi:10.1371/journal.pone.0303999)
Supplement: S1 File — (PDF) [file pone.0303999.s001.pdf]

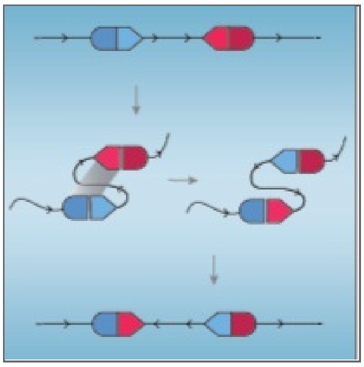

Protocols for assembly of a serine integrase-based platform for functional validation of genetic switch controllers in eukaryotic cells

Marco A. de Oliveira[1,2], Lilian H. Florentino[1,2,3], Thais T. Sales[1,2,3], Rayane N. Lima[2,3], Luciana R. C. Barros[4], Cintia G. Limia[5], Mariana S. M. Almeida[2,3], Maria L. Robledo[5], Leila M. G. Barros[2,3], Eduardo O. Melo[2,3], Daniela M. Bittencourt[2,3], Stevens K. Rehen[6,7], Martín H. Bonamino[8,9], Elibio Rech[2,3]

[1]Department of Cell Biology, Institute of Biological Science, University of Brasília, Brasília, Distrito Federal, Brazil;
[2]National Institute of Science and Technology in Synthetic Biology (INCT BioSyn), Brasília, Distrito Federal, Brazil;
[3]Embrapa Genetic Resources and Biotechnology, Brasília, Distrito Federal, Brazil;
[4]Center for Translational Research in Oncology, Instituto do Câncer do Estado de São Paulo, Hospital das Clínicas da Faculdade de Medicina de Universidade de São Paulo, São Paulo, São Paulo, Brazil;
[5]Molecular Carcinogenesis Program, Research Coordination, National Cancer Institute (INCA), Rio de Janeiro, Rio de Janeiro, Brazil;
[6]D'Or Institute for Research and Education (IDOR), Rio de Janeiro, Rio de Janeiro, Brazil;
[7]Institute of Biomedical Sciences, Federal University of Rio de Janeiro, Rio de Janeiro, Rio de Janeiro, Brazil;
[8]Cell and Gene Therapy Program, Research Coordination, National Cancer Institute (INCA), Rio de Janeiro, Rio de Janeiro, Brazil;
[9]Vice-Presidency of Research and Biological Collections (VPPCB), FIOCRUZ – Oswaldo Cruz Foundation Institute, Rio de Janeiro, Rio de Janeiro, Brazil

Elibio Rech: corresponding author: elibio.rech@embrapa.br;

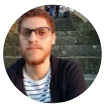

Marco Oliveira

Collection Info: Marco A. de Oliveira, Lilian H. Florentino, Thais T. Sales, Rayane N. Lima, Luciana R. C. Barros, Cintia G. Limia, Mariana S. M. Almeida, Maria L. Robledo, Leila M. G. Barros, Eduardo O. Melo, Daniela M. Bittencourt, Stevens K. Rehen, Martín H. Bonamino, Elibio Rech . Protocols for assembly of a serine integrase-based platform for functional validation of genetic switch controllers in eukaryotic cells. **protocols.io**
https://protocols.io/view/protocols-for-assembly-of-a-serine-integrase-based-c6r2zd8e

Created: Dec 21, 2023

Last Modified: Dec 23, 2023

COLLECTION integer ID: 92698

Funders Acknowledgement:

National Institute of Science and Technology in Synthetic Biology, National Council for Scientific and Technological Development
Grant ID: 465603/2014-9

Research Support Foundation of the Federal District
Grant ID: 0193.001.262/2017

ABSTRACT

This is a collection of protocols for assembly of a serine integrase-based platform for functional validation of genetic switch controllers in eukaryotic cells in human, animal and plants. Serine integrases (Ints) are a family of site-specific recombinases (SSRs) encoded by some bacteriophages to integrate their genetic material into the genome of a host. Their ability to rearrange DNA sequences in different ways including inversion, excision, or insertion with no help from endogenous molecular machinery, confers important biotechnological value as genetic editing tools with high host plasticity. Despite advances in their use in prokaryotic cells, only a few Ints are currently used as gene editors in eukaryotes, partly due to the functional loss and cytotoxicity presented by some candidates in more complex organisms. To help expand the number of Ints available for the assembly of more complex multifunctional circuits in eukaryotic cells, this protocol describes a platform for the assembly and functional screening of serine-integrase-based genetic switches designed to control gene expression by directional inversions of DNA sequence orientation. The system consists of two sets of plasmids, an effector module and a reporter module, both sets assembled with regulatory components (as promoter and terminator regions) appropriate for expression in mammals, including humans, and plants. The complete method involves plasmid design, DNA delivery, testing and both molecular and phenotypical assessment of results. This platform presents a suitable workflow for the identification and functional validation of new tools for the genetic regulation and reprogramming of organisms with importance in different fields, from medical applications to crop enhancement, as shown by the initial results obtained. This protocol can be completed in 4 weeks for mammalian cells or up to 8 weeks for plant cells, considering cell culture or plant growth time.

ATTACHMENTS

pbt9ca8tp.docx

ATTACHMENTS

pbt9ca8tp.docx

FILES

SEARCH

Protocol

NAME

Protocol for assembly of a serine integrase-based platform for functional validation of genetic switch controllers in eukaryotic cells-Human

VERSION C6RWZD7E

CREATED BY

Marco Oliveira

OPEN →

Protocol

NAME

Protocol for assembly of a serine integrase-based platform for functional validation of genetic switch controllers in eukaryotic cells-Animal

VERSION C6RYZD7W

CREATED BY

Marco Oliveira

OPEN →

Protocol

NAME

Protocol for assembly of a serine integrase-based platform for functional validation of genetic switch controllers in eukaryotic cells-Plant

VERSION C6RXZD7N

CREATED BY

Marco Oliveira

OPEN →

Protocol

NAME

MOLECULAR ANALYSES

VERSION C6RZZD76

CREATED BY

Marco Oliveira

OPEN →
