# Supplementary material for: Protocol for the establishment of a serine integrase-based platform for functional validation of genetic switch controllers in eukaryotic cells
Source: PLoS One. 2024 May 23;19(5):e0303999. doi: 10.1371/journal.pone.0303999 (PMC11115199; doi:10.1371/journal.pone.0303999)
Supplement: S2 File — (PDF) [file pone.0303999.s002.pdf]

Protocol for assembly of a serine integrase-based platform for functional validation of genetic switch controllers in eukaryotic cells-Human

In 1 collection

Marco A. de Oliveira[1,2], Lilian H. Florentino[1,2,3], Thais T. Sales[1,2,3], Rayane N. Lima[2,3], Luciana R. C. Barros[4], Cintia G. Limia[5], Mariana S. M. Almeida[2,3], Maria L. Robledo[5], Leila M. G. Barros[2,3], Eduardo O. Melo[2,3], Daniela M. Bittencourt[2,3], Stevens K. Rehen[6,7], Martín H. Bonamino[8,9], Elibio Rech[2,3]

[1]Department of Cell Biology, Institute of Biological Science, University of Brasília, Brasília, Distrito Federal, Brazil;
[2]National Institute of Science and Technology in Synthetic Biology (INCT BioSyn), Brasília, Distrito Federal, Brazil;
[3]Embrapa Genetic Resources and Biotechnology, Brasília, Distrito Federal, Brazil;
[4]Center for Translational Research in Oncology, Instituto do Câncer do Estado de São Paulo, Hospital das Clínicas da Faculdade de Medicina de Universidade de São Paulo, São Paulo, São Paulo, Brazil;
[5]Molecular Carcinogenesis Program, Research Coordination, National Cancer Institute (INCA), Rio de Janeiro, Rio de Janeiro, Brazil;
[6]D'Or Institute for Research and Education (IDOR), Rio de Janeiro, Rio de Janeiro, Brazil;
[7]Institute of Biomedical Sciences, Federal University of Rio de Janeiro, Rio de Janeiro, Rio de Janeiro, Brazil;
[8]Cell and Gene Therapy Program, Research Coordination, National Cancer Institute (INCA), Rio de Janeiro, Rio de Janeiro, Brazil;
[9]Vice-Presidency of Research and Biological Collections (VPPCB), FIOCRUZ – Oswaldo Cruz Foundation Institute, Rio de Janeiro, Rio de Janeiro, Brazil

Elibio Rech: corresponding author: elibio.rech@embrapa.br;

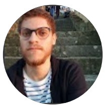

Marco Oliveira

Protocol Info: Marco A. de Oliveira, Lilian H. Florentino, Thais T. Sales, Rayane N. Lima, Luciana R. C. Barros, Cintia G. Limia, Mariana S. M. Almeida, Maria L. Robledo, Leila M. G. Barros, Eduardo O. Melo, Daniela M. Bittencourt, Stevens K. Rehen, Martín H. Bonamino, Elibio Rech . Protocol for assembly of a serine integrase-based platform for functional validation of genetic switch controllers in eukaryotic cells-Human. **protocols.io** https://protocols.io/view/protocol-for-assembly-of-a-serine-integrase-based-c6rwzd7e

Created: Dec 15, 2023

Last Modified: Dec 21, 2023

PROTOCOL integer ID: 92694

ABSTRACT

This protocol describes the assembly of a serine integrase-based platform for functional validation of genetic switch controllers in eukaryotic cells in human.

ATTACHMENTS

pbt9ca8tp.docx

MATERIALS

Biological materials

Obtaining nonactivated human T lymphocytes from PBMCs.

This protocol describes the isolation of human peripheral blood mononuclear cells (PBMCs) from leukocyte filters. Usually, 5 x 107 to 3 x 108 cells are obtained from a leukocyte filter from a healthy blood donor donation.

! CAUTION Universal precautions must be taken, experiments must be carried out in (at least) category 2 biological safety cabinets, and appropriate personal protection equipment should be used.

! CAUTION Informed consent must be obtained for the use of human blood samples.

! CAUTION Experiments with human materials must conform to all relevant institutional and governmental ethics regulations, and appropriate informed consent must be obtained for the use of human blood or patient-derived materials.

hES/NSC cell lines

Human embryonic stem (hES) cells called BR1 and human induced pluripotent stem (hiPS) cell-derived neural stem cell (NSC) lines (the cell lines were supplied by Dr. Stevens Rehen of D´Or Institute for Research and Education (iDOR), Rio de Janeiro, Brazil)

▲CRITICAL Use the BR1 cell line up to 40 passages of cells and NSCs line using up to 20 passages cells.

Reagents

Medium and supplements for PBMC

- RPMI 1640 Medium **Thermo Fisher Catalog #11875101**
- FBS **Gibco, ThermoFisher Catalog #12657-029**
- Penicillin-Streptomycin (10,000 U/mL) **Gibco - Thermo Fisher Catalog #15140122**
- L-Glutamine (200 mM) **Gibco - Thermo Fischer Catalog #25030081**

- Recombinant Human IL-2 GMP Protein, CF **R&D Systems Catalog #202-GMP-01M**

PBMC separation reagents

- PBS (Phosphate-Buffered Saline) Tablets **Thermo Fisher Catalog #003002**
- Ficoll-Paque PLUS density gradient media (GE Healthcare, cat. no. GE17-1440-02)
- Trypan Blue solution **Merck MilliporeSigma (Sigma-Aldrich) Catalog #72-57-1**
- Alcohol 70% for material sterilization
- eBioscience™ 7-AAD Viability Staining Solution **Thermo Fisher Catalog #00-6993-50**

Medium and supplements for HEK293T cell line

DMEM, powder, high glucose **Gibco - Thermo Fischer Catalog #12100046**

FBS **Gibco, ThermoFisher Catalog #12657-029**

Penicillin-Streptomycin (10,000 U/mL) **Gibco - Thermo Fisher Catalog #15140122**

Sodium Pyruvate (100 mM) **Thermo Fisher Scientific Catalog #11360070**

HEPES 1M **Thermo Fisher Scientific Catalog #15630080**

MEM Amino Acids Solution (50X) **Thermo Fisher Catalog #11130051**

MEM Non-Essential Amino Acids Solution (100X) **Thermo Fisher Catalog #11140050**

MEM Vitamin Solution (100X) **Thermo Fisher Catalog #11120052**

2-mercaptoethanol **Gibco - Thermo Fisher Catalog #21985023**

Growth medium and supplements for hECs

- mTeSR™1 500 mL Kit **STEMCELL Technologies Inc. Catalog #85850**
- Advanced™ DMEM⁄F-12 (Thermo Fisher Scientific, cat. no. 12634)
- Neurobasal® Medium and Neural Induction Supplement (NIS) (Gibco, cat. no. 12634)
- DMEM/F-12, GlutaMAX™ supplement **Thermo Fisher Catalog #10565018**

Enzymes, growth factors and chemicals for hECs

- ACCUTASE™ **STEMCELL Technologies Inc. Catalog #07920**
- ROCK Inhibitor (Y-27632) **Merck MilliporeSigma (Sigma-Aldrich) Catalog #SCM075**

Other reagents and chemicals

- HEK293T transfection reagents using HBS and CaCl2
- HEPES **Merck MilliporeSigma (Sigma-Aldrich) Catalog #H3375**
- Calcium chloride dihydrate **Merck MilliporeSigma (Sigma-Aldrich) Catalog #C5080**
- Na2HPO4 - Sodium Phosphate dibasic heptahydrate (Sigma-Aldrich, cat. no. 30413)
- Sodium chloride **Merck MilliporeSigma (Sigma-Aldrich) Catalog #S3014**
- 1S electroporation buffer:
- Potassium chloride **Merck MilliporeSigma (Sigma-Aldrich) Catalog #104936**
- Magnesium chloride hexahydrate **Merck MilliporeSigma (Sigma-Aldrich) Catalog #105833**
- di-Sodium hydrogen phosphate dodecahydrate **Merck MilliporeSigma (Sigma Aldrich) Catalog #106579**
- 120mM NaH2PO4 (VETEC, cat. no. 001236)
- 50mM Sodium Succinate (Merck, cat. no. s6638601)
- For 1SM electroporation buffer add
- 25mM Manitol (VETEC, cat. no. c000197)

▲CRITICAL Before preparing electroporation buffers, mix Na2HPO4/NaH2PO4 (phosphate buffer) and adjust the pH to 7.2 using 1 M NaOH or 1 M HCl. Use the electroporation buffer aliquots only once. Electroporation buffer aliquots (1 mL) can be stored at -20 °C for up to 3 months.

Vectors for transfection: The pT2/CAGGS-GFP plasmid was kindly provided by Dr. Sang Wang Han (UNIFESP, Brazil), and the pT3-Neo-EF1a-GFP plasmid was ordered from Addgene (Addgene, no. 69134)

Geltrex LDEV Free hESC Quality 5 ml **Thermo Fisher Scientific Catalog #A1413302**

Equipment

- Centrifuge for 50 mL tubes and microtubes (Thermo Centrifuge C3Ri, cat. no. 1115774)
- Multifunction Centrifuge (Thermo Centrifuge, Jouan B4i, cat. no.11175671)

- Biological Safety Cabinet Class 2
- Nucleofector IIb (Lonza, cat. no. AAB-1001)
- Tissue culture incubator at 5% CO_2, 21% O_2 at 37°C.
- FACSCalibur® (BD Bioscience)
- Evos XL Cell Imaging System (Thermo Fisher Scientific).

Equipment

**Corning® 75cm² U-Shaped Canted Neck Cell Culture Flask with Plug Seal Cap**	NAME
Cell Culture Flask	TYPE
Corning	BRAND
430720U	SKU
https://ecatalog.corning.com/life-sciences/b2b/UK/en/Surfaces/Advanced-Cell-Culture-Surfaces/Corning%C2%AE-and-Costar%C2%AE-Cell-Culture-Flasks/p/430720U	LINK

Equipment

**Costar® 12-well Clear TC-treated Multiple Well Plates, Bulk Pack, Sterile**	NAME
Cell culture plate	TYPE
Costar	BRAND
3512	SKU
https://ecatalog.corning.com/life-sciences/b2b/NO/en/Microplates/Assay-Microplates/96-Well-Microplates/Costar%C2%AE-Multiple-Well-Cell-Culture-Plates/p/3512	LINK

Equipment

PIPETTE, 5 ML, GRADUATED 1/10 ML, STERILE, BULK PACKAGING, 25 PCS./BAG	NAME
Sterile Syringe	TYPE
Greiner Bio-One	BRAND
606107	SKU
https://shop.gbo.com/en/netherlands/products/bioscience/liquid-handling/serological-pipettes/606107.html	LINK

Equipment

PIPETTE, 10 ML, GRADUATED 1/10 ML, STERILE	NAME
Sterile Syringe	TYPE
Greiner Bio-One	BRAND
607180	SKU
https://shop.gbo.com/en/netherlands/products/bioscience/liquid-handling/serological-pipettes/607180.html?sword_list%5B0%5D=607180%2C&no_cache=1	LINK

Equipment

PIPETTE, 25 ML, GRADUATED 2/10 ML, STERILE	NAME
Sterile Syringe	TYPE
Greiner Bio-One	BRAND
760180	SKU
https://shop.gbo.com/en/netherlands/products/bioscience/liquid-handling/serological-pipettes/760180.html?sword_list%5B0%5D=760180&no_cache=1	LINK

Equipment	
**TUBE, 50 ML, PP, 30/115 MM, CONICAL BOTTOM**	NAME
Conical Sterile Polypropylene Centrifuge Tubes	TYPE
Greiner Bio-One	BRAND
210261	SKU
https://shop.gbo.com/en/netherlands/products/bioscience/tubes-beakers/15ml-cellstar-polypropylene-tube/210261.html?sword_list%5B0%5D=210261&no_cache=1	LINK

- Sterile Polypropylene Microtubes, 1.5 mL (Greiner Bio-One, cat. no. 616275)
- Barrier Pipette tips, 10 µL, 20 µL, 200 µL, 1000 µL (KASVI cat. no. K8-10F-1, K8-20F-1K8-200F-1, K8-1000F-1)

Equipment	
**PIPETMAN L P2L, 0.2-2 µL, Metal Ejector**	NAME
Metal Ejector	TYPE
Gilson	BRAND
FA10001M	SKU
https://gb.gilson.com/GBSV/pipetman-l-p2l-0-2-2-micro-l-metal-ejector.html	LINK

Equipment	
**PIPETMAN L P10L, 0.5-10 µL, Metal Ejector**	NAME
Metal Ejector	TYPE
Gilson	BRAND
FA10002M	SKU
https://gb.gilson.com/GBSV/pipetman-l-p10l-1-10-micro-l-metal-ejector.html	LINK

Equipment	
**PIPETMAN L P200L, 20-200 µL, Metal Ejector**	NAME
Metal Ejector	TYPE
Gilson	BRAND
FA10005M	SKU
https://gb.gilson.com/GBSV/pipetman-l-p200l-20-200-micro-l-metal-ejector.html	LINK

Equipment	
**PIPETMAN L P1000L, 100-1000 µL, Metal Ejector**	NAME
Metal Ejector	TYPE
Gilson	BRAND
FA10006M	SKU
https://gb.gilson.com/GBSV/pipetman-l-p1000l-100-1000-micro-l-metal-ejector.html	LINK

PBMC electroporation

Equipment	
**Ingenio 0.2 cm Cuvettes 50 pk**	NAME
Electroporation Cuvette	TYPE
Mirus Bio	BRAND
MIR 50121	SKU
https://www.mirusbio.com/product/ingenio-electroporation-accessories-50-pk-0-2-cm-cuvettes-for-ezporator-and-lonza-nucleofector-ii-2b/	LINK

For hES/NSC cells

Equipment	
**Costar® 24-well Clear TC-treated Multiple Well Plates, Bulk Pack, Sterile**	NAME
Tissue-culture plates	TYPE
Costar	BRAND
CLS3527	SKU
https://ecatalog.corning.com/life-sciences/b2b/NL/en/Microplates/Assay-Microplates/96-Well-Microplates/Costar%C2%AE-Multiple-Well-Cell-Culture-Plates/p/3527	LINK

Equipment	
**Corning® 60 mm TC-treated Culture Dish**	NAME
Tissue-culture treated culture dishes	TYPE
Corning	BRAND
CLS430166	SKU
https://ecatalog.corning.com/life-sciences/b2b/NL/en/Surfaces/Advanced-Cell-Culture-Surfaces/Corning%C2%AE-Treated-Culture-Dishes/p/430166	LINK

Equipment	
**Corning® 100 mm TC-treated Culture Dish**	NAME
Tissue-culture treated culture dishes	TYPE
Corning	BRAND
CLS430167	SKU
https://ecatalog.corning.com/life-sciences/b2b/NL/en/Surfaces/Advanced-Cell-Culture-Surfaces/Corning%C2%AE-Treated-Culture-Dishes/p/430167	LINK

Equipment	
**Corning® Small Cell Scraper**	NAME
Cell Scraper	TYPE
Corning	BRAND
CLS3010	SKU
https://ecatalog.corning.com/life-sciences/b2b/NL/en/Cell-Culture/Cell-Culture-Accessories/Cell-Scrapers/Corning%C2%AE-Cell-Scrapers-and-Lifters/p/3010	LINK

Software

TreestarFlowJo Version 10 (http://www.flowjo.com/)

Reagent Setup

hEC/NSC cell lines

Thaw Geltrex™ Matrix solution undiluted vial at 4 °C overnight on ice. Transfer aliquots of adequate volume into Eppendorf tubes. The aliquots can be stored at −20 °C until the expiration date. Thaw aliquots on ice and dilute with 1% DMEM/F-12 and GlutaMAX™ Supplement medium.

Neural expansion medium (NEM): 50% v/v Advanced™ DMEM/F-12, 50% v/v Neurobasal® Medium, 2% Neural Induction Supplement (NIS). NEM medium can be stored at 4 °C for up to 1 month.

Prepare Y-27632 stock solution in aliquots of 10 mM in PBS. Stored at −20 °C for 1 year.

Buffer FACS for hECs/NSCs: DPBS without CaCl2 and MgCl2, 1% fetal bovine serum, fresh.

PBST (0.2% Tween 20-PBS)

Add 0.2% (vol/vol) Tween 20 to PBS. Store the buffer at room temperature for 1 year.

▲CRITICAL For a high transfection efficiency, at the electroporation step, the final volume of buffer + plasmid should be 100 µL. Do not mix less than 85 µL of buffer because it drops transfection efficiency.
▲CRITICAL Ficoll-Paque and PBS for PBMC separation must be at room temperature before use.
▲CRITICAL Antibiotic addition immediately after electroporation will cause intense cell death.

PBMC isolation ● Timing 1.5 h

25m

1 After performing the sterilization procedure in the safety cabinet, couple the syringe containing 20 mL PBS to the leukocyte filter, cut the filter tube extremity, and put it into a 50 mL tube.

Note

! CAUTION Blood can spill over from the tube, always maintain hypochlorite solution to clean up the drops.

2 Press the syringe to wash the leukocyte filter. Repeat this step if more cells are needed.

3 With a 25 mL serological pipette, add ~ 35 mL of blood at the top of the 10 mL tube containing Ficoll-Paque at Room temperature .

Note

▲CRITICAL STEP Add the blood on top of the Hystopaque very carefully. Do not take more than 20 minutes with blood on top of Ficoll; otherwise, the red blood cells will start to fall into the gradient. If the blood shakes and mixes with Ficoll-Paque, separation will not occur.
▲CRITICAL Do not shake or disturb the gradient between the Ficoll and blood.

4 Centrifuge at 800 x g, Room temperature, 00:20:00 , in a swinging-bucket rotor, without breaking 20m

Note

▲CRITICAL STEP Centrifuge must be at low acceleration and without a break setting; otherwise, the separation gradient will be lost.

5 With a 10 mL serological pipette, very carefully remove the PBMC “white ring” on top of the Ficoll-Paque and put it into a fresh tube.

Note

! CAUTION Avoid removing and placing the pipette several times as it will disturb the gradient and could contaminate the leukocytes with other fractions.
▲CRITICAL STEP Remove only the white ring containing the leukocytes without disturbing the red blood cell at the bottom. Additionally, avoid the solution above the white ring because it contains platelets and soluble factors that can change culture conditions. If the person is not experienced, add more than 10 mL of Ficoll-Paque to increase the distance between the leukocytes and the red blood cells.

6 To wash the cells, complete the volume to 50 mL with PBS and centrifuge at 400 x g, 10°C, 00:05:00 . Remove the supernatant. 5m

7 Repeat the wash step twice.

8 Resuspend the cells in 5 mL - 10 mL PBS and maintain them On ice .

9 Count the cells with a Neubauer chamber by adding Trypan blue solution at 4% for cell viability staining.

> **Note**
>
> PAUSE POINT The cells can be placed on ice for up to 2-3 hours.

PBMC electroporation ● Timing 1 h

5m

10 Add 10x10^{6} cells per electroporation reaction separating in individual microtubes.

11 Centrifuge the tubes at 400 x g, 10°C, 00:05:00 . 5m

12 Remove all supernatant with a pipette and resuspend the cells in a total of 100 µL of 1SM electroporation buffer + plasmids (integrase + target) per reaction.

13 Immediately insert the cells in the cuvette and electroporate using program U-014 on Nucleofector 2b.

Note

▲CRITICAL Electroporate and remove the cells from the cuvette quickly to avoid cell death. Wait 2 minutes for new cell electroporation.

14 Very carefully, resuspend the cells in warm RPMI/FBS 20% (no antibiotics) and plate them in total 500 µL in a 12-well plate. After 16 hours, add 500 µL RPMI/FBS 10% + P/S antibiotics + 50 U/mL IL2 + L-Glutamine.

Note

▲CRITICAL STEP If T-cell activation is needed, it should be done at this moment.

Integrase activity evaluation in PBMCs at 2 h

25m

15

Note

Integrase activity was evaluated by flow cytometry 24 h after transfection. For optimization, several time points should be evaluated. In our hands, 72 h after electroporation, eGFP expression achieved its maximum (1-35% of positive cells).

Collect the cells and place them in a microtube.

16 Centrifuge at 400 x g, 00:05:00 . Remove the supernatant. 5m

17 Wash the cells with 1 mL of cold PBS. If DNA will be extracted, separate an aliquot at this time.

18 Repeat the centrifugation 2X to wash the cells.

Note

If antibody staining will be performed, it should take place at this moment.

19 Resuspend the cells in 300 µL of cold PBS and place them in the flow cytometer tub.

Note

▲CRITICAL Fixation buffers based on formaldehyde should not be used because formaldehyde decreases GFP fluorescence.
! CAUTION Fixation buffers should not be used in case of cell viability staining with 7-AAD or PI (Propidium Iodate).

20 Add 5 µL of 7-AAD and incubate at 4 °C for 00:20:00 . 20m

Note

▲CRITICAL Keep the cells in the dark after staining. Do not wash cells after the addition of the 7-AAD staining solution.

21 Acquire at least 10,000 cells at the viable gate to evaluate eGFP expression. For a higher sensitivity, acquire more cells.

Note

▲CRITICAL STEP Acquire a nonstained GFP-negative tube as a negative control for the gating strategy. Acquire a 7-AAD -stained GFP negative tube as a negative control for GFP expression for the gating strategy. Acquire a 7-AAD -negative and GFP-positive tube (with the GFP control plasmid as pT2-GFP) for GFP-positive staining and gating strategies.

HEK293T transfection with Calcium Phosphate ● Timing 1 h

22 Plate $4x10^6$ HEK293T cells per well of a 75 cm^2 plate at least 16 h before transfection in DMEM complete medium.

Note

▲CRITICAL Cells should be less than 80% confluent, or transfection efficiency will decrease.

23 Replace the medium before transfection, adding only 10 mL of DMEM complete medium.

24 Mix the plasmids (integrase + targets, 5 µg each) to 500 µL of 2X $CaCl_2$.

Note

! CAUTION CaCl2 must be freshly prepared or maintained frozen in single-use aliquots.

25 Next, agitate $CaCl_2$ using a vortex at full speed and add 500 µL of the HBS solution drop-by-drop very slowly. Make bubbles on the solution with a Pasteur pipette. Let it rest for an instant.

Note

▲CRITICAL STEP HBS solution should be at pH 7.1 at the transfection of HEK293T cells. Any small change will have a great impact on transfection efficiency.

26 Very carefully add drop-by-drop at the cells covering all the flask with circular movements avoiding two drops at the same spot. Mix the solution with ∞ movement and place the cells in the incubator.

Integrase activity evaluation in HEK293T cells.● Timing 2h

27 Integrase activity was evaluated by flow cytometry 24 h after transfection. For optimization, several time points should be evaluated. In our hands, 72 h after electroporation, eGFP expression achieved its maximum (1-35% of positive cells).

Cell expansion of the hES cell line-Passage cells

39m

28 Culture hES cells in a 100-mm culture dish until the cells cover the dish area at 60-70% confluence. This occurs at approximately 5-6 days.

Note

▲CRITICAL Start the enzymatic dissociation with high-quality hES colonies. The spontaneous differentiation of the colonies should be removed before the split.
! CAUTION For 60-70% confluence, consider a split ratio of 1:5.

29 Prior to starting the dissociation, coat the fresh 100-mm culture dishes with 6 mL/dish of cold Geltre 30m and place it into the incubator at 37 °C for at least 00:30:00 .

30 Remove Geltrex™, and immediately add 9 mL/dish of fresh growth mTeSR™ 1 medium to prevent drying.

31 Place the fresh culture dishes into the incubator at 37 °C .

32 Carefully aspirate the old growth medium from the cell culture dish.

33 Wash the dish with 4 mL of DPBS without calcium and magnesium (DPBS-/-) and remove immediately.

34 Add 4 mL of Accutase™ into the 100-mm culture dish, ensure that cover of dish area.

35 Incubate the cell suspension at 37 °C for 00:05:00 . 5m

Note

! CAUTION Monitoring the cell viability and morphology changes, it should be observed cells round up into the colonies while remaining attached to the surface of the dish. If necessary, repeat step 35.
! CAUTION Dissociate hES cells into small clusters rather than single-cell suspensions at each passage.

36 Add 3 mL of DMEM/F12 and gently triturate the clusters across the dish surface.

37 Scrape the attached cells using a cell scraper.

38 Transfer the unattached cell suspension into a 15 mL conical tube containing 3 mL of DMEM/F12 using a 5 mL serological pipette.

39 Spin down at 100 x g, 25°C, 00:04:00 . 4m

40 Aspirate and discard the supernatant.

41 Resuspend the cell pellet with 5 mL of fresh growth mTeSR™ 1 Medium using a 5 mL serological pipette.

42 Add the cell suspension 1 mL/dish into fresh 100-mm culture dishes prepared previously at step 28-31.

43 Incubate the cells at 37 °C , 95% O2, 5% CO_2 in humidified air.

44 After 24 h, replace the old growth medium with fresh mTeSR™ 1 medium.

45 Split the cells every 5-6 days.

Maintenance of hES cell culture ● Timing 5 min daily

46 Carefully aspirate the old growth medium from the cell culture dish.

47 Replace with 10 mL of fresh growth mTeSR™ 1 Medium.

48 Incubate the cells at 37 °C, 95% O2, and 5% CO_2 in humidified air until 60-70% confluent.

49 Replace the medium daily.

Note

! CAUTION After 10 passages to ensure the pluripotency status and genetic integrity of hES cells

Cells preparation for electroporation ● Timing 6 d

50 Replace the old growth mTeSR™1 Medium for 6 days.

Note

! CAUTION After 80% confluence, the number of cells/dish should be increased to approximately 8-10 x 106, sufficient for 8-10 electroporation reactions.

hES cell electroporation ● Timing 1 h

51 (Optional) Prior to starting dissociation, add iROCK ([M] 10 micromolar (µM)) in the 100-mm cell culture dish for 01:00:00. 1h

52 Coat the fresh 12-well plate with 6 mL of cold Geltrex™ (0.5 µL/well) as described in steps 29-31.

53 Aspirate Geltrex™ and add 0.5 µL/well of antibiotic-free fresh mTeSR™1 Medium with iROCK ([M] 10 micromolar (µM)).

54 Carefully aspirate the old growth medium from the cell 100-mm culture dishes.

55 Wash with 4 mL/dish of DPBS-/- and remove immediately.

56 Add 4 mL /dish of Accutase™, ensure that cover of dish area.

57 Incubate the cell suspension at 37 °C for 5-10 minutes.

Note

! CAUTION The spontaneous differentiation of the colonies should be removed before the split.
! CAUTION During the incubation with Accutase**TM** monitoring the morphology of colonies, it should be observed separated round cells by microscopy. Dissociate hES cells into single-cell suspensions and avoid clusters. If necessary, repeat step 30.
▲CRITICAL STEP High transfection efficiency is dependent on high-quality hES colonies as well as efficient single-cell enzymatic dissociation.

58 Add 3 mL /dish of DMEM/F12 and scrape the attached cells using a cell scraper.

59 Gently triturate the clusters across dish surface.

60 Transfer the unattached cell suspension using a 5 mL serological pipette into a 50 mL conical tube containing 10 mL of DMEM/F12.

61 Spin down at 100 x g, 25°C, 00:04:00. 4m

62 Remove the supernatant and wash with 10 mL of DPBS-/-.

63 Count the cells with Trypan blue dye exclusion.

Note

▲CRITICAL STEP An ineffective transfection is observed using lower cell density or low cell viability.

64 Spin down at 100 x g, 25°C, 00:04:00. 4m

65 Aspirate and discard the supernatant.

Note

▲CRITICAL STEP Calculate the total volume needed to resuspend the hES cells. A single electroporation reaction requires 1x106 viable hES cells. It should be transferred 2 mL per electroporation reaction (i.e., For 3 electroporation reactions, add a total volume of 6 mL into a tube.)

66 Resuspend the cells with ice-cold DPBS-/-.

67 Gently mix the suspension cells and transfer 2 mL/15 mL conical tube ($1x10^6$ cells per tube).

68 Spin down at 100 x g, 4°C, 00:04:00 . 4m

69 Remove the supernatant and resuspend the pellet with 1SM electroporation buffer mix with the plasmids in a total volume of 100 µL .

Note

▲CRITICAL STEP For transient expression, hES cells can be electroporated with 12 µg of pIE plasmids and 8 µg of pSG.

70 Immediately transfer the suspension cells into the 0.2 cm electroporation cuvette, preventing bubbles.

71 Electroporate the cells by using the Nucleofector 2B program A-23.

Note

▲CRITICAL STEP Electroporation frequently leaves considerable cell death.

72 Immediately add 1 mL of fresh mTeSR™1 Medium with iROCK ([M] 10 micromolar (µM)) into the electroporation cuvette using a micropipette with a 1000 µL tip to avoid low cell viability.

73 Immediately transfer the cell suspension into a 15 mL conical tube containing 0.5 mL of antibiotic-free fresh mTeSR™1 Medium with iROCK ([M] 10 micromolar (µM)).

Note

▲CRITICAL STEP Repeat steps 69-72 for each single electroporation reaction. It is important to transfect one reaction at a time. A long period of cell incubation with the electroporation buffer can affect cell viability.

74 Gently mix the cell suspension and transfer into three wells (0.5 mL/well) of the coated Geltrex™ 12-well plate previously prepared in steps 51-52.

75 Incubate the cells at 37 °C, 95% O2, 5% CO_2 in humidified air.

76 After 24 h, replace the old growth medium with antibiotic-free fresh mTeSR™1 medium.

Postelectroporation ● Timing 2 d

77 After 48 h of transfection, evaluate the integrase activity by flow cytometry.

Preparation of sample and flow cytometry ● Timing 2 h

78 Harvest cells using Accutase™ as described in step 24.

79 Remove the old growth medium and wash with 0.5 mL/well of DPBS-/-, remove immediately.

80 Add 0.4 mL/well of Accutase™.

81 Incubate the cells in the incubator at 37 °C for 5-10 minutes.

Note

! CAUTION Monitoring the morphology of colonies, it should be observed individual round cells by microscopy. Dissociate hES cells into single-cell suspensions, avoiding clusters. If necessary, repeat step 81.

82 Add 0.5 mL /well of DMEM/F12 into a 15 mL conical tube containing 5 mL of DMEM/F12.

83 Spin down at 100 x g, 25°C, 00:04:00 . 4m

84 Aspirate and discard the supernatant.

85 Wash the cells with 2 mL of ice-cold DPBS-/-.

86 Spin at 100 x g, 4°C, 00:04:00 . 4m

87 Remove the supernatant and resuspend the cells in 2 mL of ice-cold DPBS-/-.

88 Count the cells with Trypan blue dye exclusion.

89 Transfer 1 x 10^5 cells/tube for flow cytometry analysis.

90 Keep the cell suspension in the original 15 mL conical tube and collect the pellet for DNA extraction.

91 Spin at 100 x g, 4°C, 00:04:00 . 4m

92 Resuspend the cells (1 x 10^5 cells/tube) in 300 µL of ice-cold buffer for FACS.

93 Add 5 µL of 7-AAD into a tube with the cells and incubate at Room temperature for 00:10:00 . 10m

Note

▲CRITICAL STEP After incubation, store the tubes at 4 °C protected from light prior to analysis.
▲CRITICAL STEP Acquire at least 10,000 events at the viable gate to evaluate eGFP expression.

Cell expansion of the NSC cell line ● Timing 14-20 d-Passage ...

94 Culture NSC cells into a 60-mm culture dish until the cells cover the dish area, at 85-90% confluence. It is approximately 6-7 days.

Note

▲CRITICAL STEP Start enzymatic dissociation with high-quality NSC cells.
▲CRITICAL STEP For over confluence consider a split ratio of 1:8 to 1:10. NSCs can be seeded at a density of 0.5x105 cells/cm2. For the 60-mm culture dish, consider an area of 20 cm2.

95 Prior to starting the dissociation, coat the fresh 60-mm culture dishes with 3 mL/dish of cold Geltrex 30m

and place it into the incubator at 37 °C for at least 00:30:00 .

96 Remove Geltrex™, and immediately add 2 mL /dish of fresh NEM medium to prevent drying.

97 Place fresh culture dishes into the incubator at 37 °C .

98 Carefully aspirate the old medium from the cell culture dish.

99 Wash the dish with 2 mL of DPBS-/-, remove immediately.

00 Add 2 mL of Accutase™ into the 60-mm culture dish, ensure that cover of dish area.

01 Incubate the cell suspension at 37 °C for 3-5 minutes.

Note

▲CRITICAL STEP Monitoring the cell viability and morphology changes, it should be observed that cells round up while remaining attached to the surface of the dish. If necessary, repeat step 101.
▲CRITICAL STEP Dissociation of NSC cells into single-cell suspensions is required at each passage.

02 Add 3 mL of DMEM/F12 and gently triturate the clusters across the dish surface.

03 Transfer the unattached cell suspension into a 15 mL conical tube containing 3 mL of DMEM/F12 using a 5 mL serological pipette.

04 Spin down at 300 x g, 25°C, 00:04:00. 4m

05 Aspirate and discard the supernatant.

06 Wash with 5 mL of DMEM/F12 using a 5 mL serological pipette.

07 Count the cells with Trypan blue dye exclusion.

08 Spin down at 300 x g, 25°C, 00:04:00. 4m

09 Resuspend the cell pellet with fresh growth NEM medium using a 5 mL serological pipette.

Note

! CAUTION Calculate the total volume needed to resuspend the NSC cells. Consider seed 1x106 cells/dish in 1 mL.

10 Add the cell suspension at 1 mL/dish into fresh 60-mm culture dishes previously prepared at steps 94-97. Should have a total volume of 3 mL/dish.

11 Incubate the cells at 37 °C, 95% O2, 5% CO_2 in humidified air.

12 After 24 h, replace the old growth medium with 3 mL of fresh NEM medium.

13 Split the cells every 6 days.

Maintenance of NSC cell culture for 5 min daily

14 Carefully aspirate the old growth medium from the cell culture dish.

15 Replace with 4 mL of fresh growth NEM medium.

16 Incubate the cells at 37 °C, 95% O2, and 5% CO_2 in humidified air until they reach 80-90% confluent.

17 Change the growth medium every 2 days.

Note

▲CRITICAL STEP After 10 passages to ensure the pluripotency status and genetic integrity of NSC cells

Cells preparation for electroporation ● Timing 6 d

18 Replace the old growth NEM media for 6 days.

Note

! CAUTION After 90-95% confluence, the number of cells/dish should be increased to approximately 8-12 x 10^6, sufficient for 8-12 electroporation reactions.

NSC cell electroporation ● Timing 1 h

19 (Optional) Prior to starting dissociation, add iROCK ([M] 10 micromolar (µM)) into 60-mm culture cell dish for 01:00:00 . 1h

20 Coat the fresh 12-well plate with 6 mL of cold Geltrex™ (0.5 µL/well) as described in steps 95-97.

21 Aspirate Geltrex™ and add 0.5 µL /well of antibiotic-free fresh NEM medium with iROCK ([M] 10 micromolar (µM)).

22 Carefully aspirate the old growth medium from the cell 60-mm culture dishes.

23 Wash with 2 mL /dish of DPBS-/-, and remove immediately.

24 Add 2 mL /dish of Accutase™, ensure that cover of dish area.

25 Incubate the cell suspension at 37 °C for 3-5 minutes.

Note

! CAUTION Discard the cells if spontaneous differentiation is observed.
▲CRITICAL STEP High transfection efficiency is dependent on high-quality NSCs as well as efficient single-cell enzymatic dissociation.

▲CRITICAL STEP During incubation with Accutase^{TM} monitor the morphology of colonies by microscopy. Cells must be rounded. Dissociate NSC cells into single-cell suspensions. If necessary, repeat step 125.
▲CRITICAL STEP Ineffective transfection is observed in the presence of cluster cells.

26 Add 2 mL /dish of DMEM/F12 and gently triturate the clusters across the dish surface.

27 Remove unattached cells using a 5 mL serological pipette.

28 Transfer the cell suspension into a 50 mL conical tube containing 10 mL of DMEM/F12.

29 Spin down at 300 x g, 25°C, 00:04:00 . 4m

30 Remove the supernatant and wash with 5 mL of DPBS-/-.

31 Count the cells with Trypan blue dye exclusion.

32 Spin down at 300 x g, 25°C, 00:04:00 . 4m

Note

! CAUTION Calculate the total volume needed to resuspend the NSC cells. A single electroporation reaction requires 1x106 viable NSCs. It should be transferred 2 mL per electroporation reaction (i.e., For 3 electroporation reactions, add a total volume of 6 mL into a tube.)
▲CRITICAL STEP An ineffective transfection is observed using lower cell density or low cell viability.

33 Resuspend the cells with ice-cold DPBS-/-.

34 Gently mix the suspension cells and transfer 2 mL/15 mL conical tube.

35 Spin down at 300 x g, 4°C, 00:04:00 . 4m

36 Remove the supernatant and resuspend the cells with 1S electroporation buffer mix with the plasmids in a total volume of 100 µL .

Note

▲CRITICAL STEP For transient expression, NSC cells can be electroporated with 12 µg of pIE plasmids and 8 µg of pSG.

37 Immediately transfer the suspension cells into the 0.2 cm electroporation cuvette, avoid bubbles.

38 Electroporate the cells by using the Nucleofector 2D program A-33.

Note

! CAUTION Cell death is frequently observed after electroporation.

39 Immediately add 1 mL of fresh NEM media with iROCK ([M] 10 micromolar (µM)) into the electroporation cuvette using a micropipette with a 1000 µL tip to avoid low cell viability.

40 Immediately transfer the cell suspension into a 15 mL conical tube containing 0.5 mL of antibiotic-free fresh NEM media with iROCK ([M] 10 micromolar (µM)).

Note

▲CRITICAL STEP Repeat steps 136-138 for each single electroporation reaction. It is important to transfect one reaction for a specified time. A long period of cell incubation with the electroporation buffer can affect cell viability.

41 Gently mix the cell suspension and transfer into three wells (0.5 mL/well) of the coated Geltrex™ 12-well plate previously prepared in step 28.

42 Incubate the cells at 37 °C, 95% O2, 5% CO_2 in humidified air.

43 After 24 h, replace the old growth medium with antibiotic-free fresh NEM medium.

Note

? TROUBLESHOOTING

Postelectroporation ● Timing 2 d

44 After 48 h of transfection, the integrase activity can be evaluated by using flow cytometry.

Preparation of sample and flow cytometry ● Timing 2 h

22m

45 Harvest cells using Accutase™.

46 Remove the old growth medium and wash with 0.5 mL/well of DPBS-/-, remove immediately.

47 Add 0.4 mL /well of Accutase™.

48 Incubate the cells in the incubator at 37 °C for 5-10 minutes.

Note

▲CRITICAL STEP Monitor the morphology of colonies by microscopy. Rounded cells must be observed.

49 Add 0.5 mL /well of DMEM/F12 into a 15 mL conical tube containing 5 mL of DMEM/F12.

50 Spin down at 300 x g, 25°C, 00:04:00 . 4m

51 Wash the cells with ice-cold DPBS-/-.

52 Spin at 300 x g, 4°C, 00:04:00 . 4m

53 Remove the supernatant and resuspend the cells in 2 mL of ice-cold DPBS-/-.

54 Count the cells with Trypan blue dye exclusion.

55 Transfer 1 x 10^5 cells/tube by flow cytometry analyses.

56 Keep the cell suspension in the original 15 mL conical tube and collect the pellet for DNA extraction.

57 Spin at 300 x g, 4°C, 00:04:00 . 4m

58 Resuspend the cells (1 x 10^5 cells/tube) in 300 µL of ice-cold buffer for FACS.

59 Add 5 µL of 7-AAD into a tube with the cells and incubate at Room temperature for 00:10:00 . 10m

Note

▲CRITICAL STEP After incubation, store the tubes at 4 °C protected from light prior to analysis.
▲CRITICAL STEP Acquire at least 10,000 events at the viable gate to evaluate eGFP expression.
