# Supplementary material for: Protocol for the establishment of a serine integrase-based platform for functional validation of genetic switch controllers in eukaryotic cells
Source: PLoS One. 2024 May 23;19(5):e0303999. doi: 10.1371/journal.pone.0303999 (PMC11115199; doi:10.1371/journal.pone.0303999)
Supplement: S3 File — (PDF) [file pone.0303999.s003.pdf]

Protocol for assembly of a serine integrase-based platform for functional validation of genetic switch controllers in eukaryotic cells-Animal

In 1 collection

Marco A. de Oliveira[1,2], Lilian H. Florentino[1,2,3], Thais T. Sales[1,2,3], Rayane N. Lima[2,3], Luciana R. C. Barros[4], Cintia G. Limia[5], Mariana S. M. Almeida[2,3], Maria L. Robledo[5], Leila M. G. Barros[2,3], Eduardo O. Melo[2,3], Daniela M. Bittencourt[2,3], Stevens K. Rehen[6,7], Martín H. Bonamino[8,9], Elibio Rech[2,3]

[1]Department of Cell Biology, Institute of Biological Science, University of Brasília, Brasília, Distrito Federal, Brazil;
[2]National Institute of Science and Technology in Synthetic Biology (INCT BioSyn), Brasília, Distrito Federal, Brazil;
[3]Embrapa Genetic Resources and Biotechnology, Brasília, Distrito Federal, Brazil;
[4]Center for Translational Research in Oncology, Instituto do Câncer do Estado de São Paulo, Hospital das Clínicas da Faculdade de Medicina de Universidade de São Paulo, São Paulo, São Paulo, Brazil;
[5]Molecular Carcinogenesis Program, Research Coordination, National Cancer Institute (INCA), Rio de Janeiro, Rio de Janeiro, Brazil;
[6]6 D'Or Institute for Research and Education (IDOR), Rio de Janeiro, Rio de Janeiro, Brazil;
[7]Institute of Biomedical Sciences, Federal University of Rio de Janeiro, Rio de Janeiro, Rio de Janeiro, Brazil;
[8]Cell and Gene Therapy Program, Research Coordination, National Cancer Institute (INCA), Rio de Janeiro, Rio de Janeiro, Brazil;
[9]Vice-Presidency of Research and BiologicCollections (VPPCB), FIOCRUZ – Oswaldo Cruz Foundation Institute, Rio de Janeiro, Rio de Janeiro, Brazil

Elibio Rech: corresponding author: elibio.rech@embrapa.br;

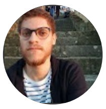

Marco Oliveira

Protocol Info: Marco A. de Oliveira, Lilian H. Florentino, Thais T. Sales, Rayane N. Lima, Luciana R. C. Barros, Cintia G. Limia, Mariana S. M. Almeida, Maria L. Robledo, Leila M. G. Barros, Eduardo O. Melo, Daniela M. Bittencourt, Stevens K. Rehen, Martín H. Bonamino, Elibio Rech . Protocol for assembly of a serine integrase-based platform for functional validation of genetic switch controllers in eukaryotic cells-Animal. **protocols.io** https://protocols.io/view/protocol-for-assembly-of-a-serine-integrase-based-c6ryzd7w

Created: Dec 20, 2023

Last Modified: Dec 23, 2023

PROTOCOL integer ID: 92696

ABSTRACT

This protocol details the assembly of a serine integrase-based platform for functional validation of genetic switch controllers in eukaryotic cells in animal.

ATTACHMENTS

pbt9ca8tp.docx

MATERIALS

Biological materials

Bovine fibroblast (see 'Reagent setup') cells were isolated according to the protocol described by Freshney[51], with modifications.

Reagents

- Growth medium and supplements
- distilled water, dH2O, sterile
- DMEM (Dulbecco's Modified Eagle Medium,) powder, low glucose (Gibco, cat. no.3600-034)
- Sodium bicarbonate Merck MilliporeSigma (SigmaAldrich) Catalog #S5761
- Glucose Merck MilliporeSigma (SigmaAldrich) Catalog #G7021
- L-Glutamine Merck MilliporeSigma (SigmaAldrich) Catalog #G6392
- Sodium Pyruvate Merck MilliporeSigma (SigmaAldrich) Catalog #P5280
- FBS Gibco, ThermoFisher Catalog #12657-029
- Penicillin G sodium salt Merck MilliporeSigma (SigmaAldrich) Catalog #P3032-25MU
- Streptomycin sulfate salt Merck MilliporeSigma (SigmaAldrich) Catalog #S9137
- Phosphate buffered saline Merck MilliporeSigma (SigmaAldrich) Catalog #P4417-50TAB
- Trypsin-EDTA (0.05%), phenol red Thermo Fisher Catalog #25300062
- Trypan Blue solution Merck MilliporeSigma (SigmaAldrich) Catalog #T8154
- Alcohol 70% for material sterilization (any brand)
  ! CAUTION alcohol is flammable. Handle it in a fume hood with proper PPE.

Transfection

- Lipofectamine™ LTX Reagent with PLUS™ Reagent Thermo Fisher Catalog #15338030
- Opti-MEM™ I Reduced Serum Medium Thermo Fisher Catalog #31985070

Flow cytometry

- Rinse: Deionized water
- Phosphate buffered saline Merck MilliporeSigma (SigmaAldrich) Catalog #P4417-50TAB
- Isopropanol Merck MilliporeSigma (SigmaAldrich) Catalog #563935
- Sterilizer solution: 0.4-0.7%Hypochlorite
- COULTER CLENZ AGENT (500ML) Beckman Coulter Catalog #8546929
- Flow Sight Calibration Beads (Amnis, cat. no. 400300)

Viability assay

- Phosphate buffered saline Merck MilliporeSigma (SigmaAldrich) Catalog #P4417-50TAB
- Dimethyl sulfoxide (DMSO) Merck MilliporeSigma (SigmaAldrich) Catalog #D2650
- MTT (3-(4,5-Dimethylthiazol-2-yl)-2,5-Diphenyltetrazolium Bromide) Thermo Fisher Catalog #M6494

Plasmid extraction and cloning/PCR

- LB Broth (Lennox) Merck MilliporeSigma (SigmaAldrich) Catalog #L3022
- One Shot™ MAX Efficiency™ DH10B T1 Phage-Resistant Cells Thermo Fisher Catalog #12331013
- dH 2 O, sterile
- Ampicillin sodium salt Merck MilliporeSigma (SigmaAldrich) Catalog #A0166
- Kanamycin monosulfate Merck MilliporeSigma (SigmaAldrich) Catalog #K1377
- QIAGEN Plasmid Maxi Kit (25) Qiagen Catalog #12163
- pGEM(R)-T Easy Vector System I Promega Catalog #A1360
- EcoRI - 10,000 units New England Biolabs Catalog #R0101S
- Platinum™ Taq DNA Polymerase Thermo Fisher Catalog #10966018
- PureYield(TM) Plasmid Miniprep System, 250 preps Promega Catalog #A1222
- ReliaPrep™ DNA Clean-Up and Concentration System Promega Catalog #A2892
- Agarose Merck MilliporeSigma (SigmaAldrich) Catalog #A9539
- SYBR Thermo Fisher Scientific Catalog #S33102
- 1 Kb Plus DNA Ladder Invitrogen - Thermo Fisher Catalog #10787018
- QIAgen DNeasy Blood and Tissue Kit, 50 rxn Qiagen Catalog #69504

Equipment

- Equipment for bovine cell isolation, culture and analysis
- Biological Safety Cabinet Class 2 (VecoFlow LTDA)
- Stericup Quick Release-GP Sterile Vacuum Filtration System 500 mL (Millipore, cat. no.S2GPU05RE)
- 0.22 µm PES membrane (KASVI, cat. no. K18-230 or equivalent)
- Vacuum pump (Millipore, model WP6111560)
- Bottles with screw cap Boro 3.3 500 mL and 1L (Boeco Germany, cat. no. BOE5080445, BOE5080545)
- Graduated cylinder 500 mL (any brand)
- Beaker 500 mL (any brand)
- Pipette tips, 10 µL, 200 µL, 1000 µL (any brand)

Equipment	
**PIPETMAN L P2L, 0.2-2 µL, Metal Ejector**	NAME
Metal Ejector	TYPE
Gilson	BRAND
FA10001M	SKU
https://gb.gilson.com/GBSV/pipetman-l-p2l-0-2-2-micro-l-metal-ejector.html	LINK

Equipment	
**PIPETMAN L P10L, 0.5-10 µL, Metal Ejector**	NAME
Metal Ejector	TYPE
Gilson	BRAND
FA10002M	SKU
https://gb.gilson.com/GBSV/pipetman-l-p10l-1-10-micro-l-metal-ejector.html	LINK

Equipment	
**PIPETMAN L P200L, 20-200 µL, Metal Ejector**	NAME
Metal Ejector	TYPE
Gilson	BRAND
FA10005M	SKU
https://gb.gilson.com/GBSV/pipetman-l-p200l-20-200-micro-l-metal-ejector.html	LINK

Equipment	
**PIPETMAN L P1000L, 100-1000 µL, Metal Ejector**	NAME
Metal Ejector	TYPE
Gilson	BRAND
FA10006M	SKU
https://gb.gilson.com/GBSV/pipetman-l-p1000l-100-1000-micro-l-metal-ejector.html	LINK

Semi-Micro Analytical Balance (Shimadzu, model AUW220D)

Equipment	
**Orion Star™ A211 Benchtop pH Meter**	NAME
pH Meter	TYPE
Thermo Scientific	BRAND
STARA2119	SKU
https://www.thermofisher.com/order/catalog/product/STARA2119	LINK

- Water bath (Ultronic, model Q3.0/040A or equivalent)
- Cell culture flask, 25 and 75 cm2 (TPP, cat. no. 90025, 90075)
- Flat-bottom cell culture plates, 24 and 96-well (TPP, cat. no. 92024, 92096)
- LoBind Microcentrifuge Tubes, 1.5 mL and 2.0 mL (Axygen, cat. no. MCT-150-L-C, MCT-200-L-C)
- Cell incubator (37°C, 5% CO2) (Thermo Scientific)
- Axiovert 135M fluorescence microscope (Carl Zeiss)
- Neubauer chamber (KASVI, cat. no. K5-0111)
- NanoDrop 2000c Spectrophotometer (Thermo Scientific)
- Conical centrifuge tube, 15 mL and 50 mL (Kasvi, cat. no. K19-0015 and K19-0050 respectively)
- Mini spin mini centrifuge for microtubes (Eppendorf, MFG Part Number, 22620100)

- Erlenmeyer flask, glass 250 and 500 mL (any brand)
- Parafilm® M (Bemis, cat. no. P6543)
- Thermocycler with programmable temperature control, 96 wells T100™ Thermal Cycler (Bio-Rad, cat. no.1861096)
- Electrophoresis PowerPac (Bio-Rad)
- SmartView Pro 1100 Imager System (Major Science, cat. no. UVCI-1100)
- Sunrise Microplate Reader (Tecan)
- Amnis® brand FlowSight® Imaging Flow Cytometer (Merck Millipore, cat. no. 100300 or equivalent)
- Ultrospec10 cell density meter spectrophotometer (Amersham Biosciences)

Software

Magellan https://www.selectscience.net/products/magellan-data-analysis-software/?prodID=20443
IDEAS software https://www.merckmillipore.com/BR/pt/20150121_204850?ReferrerURL=https%3A%2F%2Fwww.google.com%2F
SnapGene
https://www.snapgene.com/support/downloads

Reagent Setup

Bovine fibroblast isolation

Bovine fibroblast cells were isolated according to the protocol described by Freshney51 with modifications. The cells were removed from 14-month-old Nelore (Bos indicus) bull oxtail by biopsies and washed three times in 0.05% trypsin (Gibco). The cells were then transferred to 25-cm2 cell culture flasks and incubated in DMEM (Gibco) supplemented with 10% FCS (Gibco) and penicillin–streptomycin at 37 °C in a 5% CO2 atmosphere. After three passages or when the fibroblast cultures showed homogeneity, the cells were ready for transfection. Cell cultures with 60% to 70% confluence were picked. ! CAUTION The use of bovine cells was performed under ethical guidelines and was approved by the Ethics Committee on the Use of Animals (CEUA) of Embrapa Genetic Resources and Biotechnology in March 2013 approval reference no. 001/2013.

Culture medium stock

For 1 L of DMEM, measure approximately 90% of the volume to be prepared with sterile distilled water (15-20 °C), and slowly add the 13.5 g of DMEM powder under constant agitation at room temperature. After completely dissolving the DMEM powder, add 3.7 g of sodium bicarbonate (NaHCO3) per liter, complete with sterile distilled water up to 1 L and store at 4 °C for up to 6 months.

Culture medium (working solution)

For each 400 mL of DMEM solution, add 40 mL of FBS (10% (vol/vol) final), 0.4 mL of penicillin/streptomycin (1% (vol/vol) final), 44 mg of 100X sodium pyruvate, xx mg of D-(+)-glucose, and 116 mg of L-glutamine 200 mM. Filter through a 0.22 µm Vacuum Filtration System and store at 4 °C for up to 2 months.

MTT stock solution

Dissolve 500 mg of MTT powder in 10 mL of 1X PBS (pH 3.7), stir the solution for approximately 1 hour in the dark, sterilize it by filtration with a 0.22 µm filter ▲CRITICAL STEP store 10 mL aliquots (50 mg/mL) at -20 °C in the dark.

Cell Passage ● Timing 30 min

1 Cultivate bovine fibroblast cells at 5.0×10^5 cells per culture flask with a 75 cm2 surface area. Well area coverage, between 60-80% confluence. It takes approximately 8-10 days.

2 Before starting dissociation, place the working aliquot of 0.05% trypsin and EDTA solution in the incubator at 37 °C for at least 00:10:00 . 10m

3 Remove the DMEM culture medium and wash with Dulbecco's Phosphate-Buffered Saline (DPBS, GIBCO). Discard the DPBS and immediately add 2 mL of 0.05% trypsin and EDTA. Make sure to cover the plate area.

4 Place the culture flask in the incubator at 37 °C for 00:05:00 . 5m

Note

▲CRITICAL STEP Monitoring cell viability and morphology changes, round cells should be observed while remaining adhered to the plate surface. If necessary, repeat step 4.
▲CRITICAL STEP Cells need to be dissociated in each passage.

5 Add 2 mL of DMEM.

6 Transfer the loose cell suspension to a 1.5 or 2 mL centrifugation tube.

7 Spin at 1300 rpm, Room temperature, 00:05:00 . 5m

8 Aspirate and discard the supernatant.

9 Wash with 5 mL of DPBS using a serological pipette.

10 Spin at 1300 rpm, Room temperature, 00:05:00 . 5m

11 Aspirate and discard the supernatant.

12 Resuspend the cell pellet with fresh DMEM medium using a serological pipette.

13 Add 1 mL of cells/flask culture to new culture flasks with a 75 cm^2 surface area that have been previously prepared.

Note

Should have a total volume of 10 mL.

14 Incubate the cells at 37 °C, 95% humidity, 5% CO_2.

15 After 24h, replace the old culture medium with 10 mL of fresh DMEM medium.

Note

▲CRITICAL STEP Regularly observe the cells to ensure they are growing properly and that there is no contamination.
▲CRITICAL STEP When the cells reach confluence, subculturing is necessary to avoid high cell density.

16 Divide the cells every 5 days.

Bovine fibroblast cell transfection with Lipofectamine LTX an...

17 Prepare the cells for transfection: the cells should be at a specific stage of culture and in the appropriate medium for transfection.

18 Prepare the transfection solution: mix the LTX Plus with the desired plasmid following the manufacturer's instructions.

19 Add the transfection solution to the cells: add the transfection solution to cells grown in culture plate or to the medium for suspended cells.

20 Incubate the cells: incubate the cells with the transfection solution for a specific period of time, usually 4-6 hours.

Note

▲CRITICAL Add new culture medium: after the incubation period, change the culture medium to remove the transfection solution and allow the cells to continue developing.
▲CRITICAL STEP Integrase activity can be evaluated by flow cytometry 24 hours after transfection. For optimization, several time points should be evaluated. In our hands, 72 hours after transfection eGFP expression was reduced.
▲CRITICAL STEP The results were analyzed 48 h after transient transfection by flow cytometry.

Analysis of integrase expression in bovine fibroblast cells by ...

21 Dissociating cells using 0.05% Trypsin-EDTA (1X):

21.1 Remove the old growth medium from each well and wash with 0.5 mL/well of DPBS, remove immediately.

21.2 Add 0.5 mL /well of 0.05% Trypsin-EDTA.

21.3 Incubate the cells in the incubator at 37 °C for 00:05:00 . 5m

Note

▲CRITICAL STEP Monitoring colony morphology, rounded cells should be observed by microscopy.

21.4 Add 0.5 mL /well of DMEM in a microcentrifuge and 1.5 or 2 mL tube containing DMEM.

21.5 Spin at 1300 rpm, 00:05:00 . 5m

21.6 Wash the cells with DPBS.

21.7 Spin at 1300 rpm, 00:05:00 . 5m

21.8 Remove the supernatant and resuspend the cells with DPBS.

21.9 Transfer the cells to a 1.5 mL centrifugation tube for flow cytometry analysis.

Note

▲CRITICAL STEP Store the tubes at 37°C protected from light before analysis.
▲CRITICAL STEP Acquire at least 10,000 events in the viable gate to evaluate eGFP expression.

Cell viability assays. ● Timing 7h

1d 4h

22 Count the cells with a Neubauer chamber and cultivate bovine fibroblasts and HEK 293T cells in 96-well plates at a density of 1x105 cells/well in triplicate and grown for 24:00:00 at 37 °C in a 5% CO_2 atmosphere. 1d

23 After 48 hours of transient cotransfection, incubate the cells with 15 µL of 3-(4,5-dimethylthiazol-2-yl)-2,5-diphenyltetrazolium bromide (MTT; Thermo Fisher Scientific) (5 mg/mL).

Note

▲CRITICAL Protect from light!
▲CRITICAL For the negative control, use 20 µL of dimethyl sulfoxide (DMSO) directly on the cells, in a final volume of 200 µL per well.

24 Incubate for 04:00:00 at 37 °C. 4h

25 Remove the MTT solution and add 150 µL of DMSO to each well to dissolve formazan crystals.

Note

▲CRITICAL perform the procedure in the absence of light and do not aspirate the crystals.

26 Read the absorbance at 595 nm in a plate reader or spectrophotometer.
