# Supplementary material for: Protocol for the establishment of a serine integrase-based platform for functional validation of genetic switch controllers in eukaryotic cells
Source: PLoS One. 2024 May 23;19(5):e0303999. doi: 10.1371/journal.pone.0303999 (PMC11115199; doi:10.1371/journal.pone.0303999)
Supplement: S4 File — (PDF) [file pone.0303999.s004.pdf]

Protocol for assembly of a serine integrase-based platform for functional validation of genetic switch controllers in eukaryotic cells-Plant

In 1 collection

Marco A. de Oliveira[1,2], Lilian H. Florentino[1,2,3], Thais T. Sales[1,2,3], Rayane N. Lima[2,3], Luciana R. C. Barros[4], Cintia G. Limia[5], Mariana S. M. Almeida[2,3], Maria L. Robledo[5], Leila M. G. Barros[2,3], Eduardo O. Melo[2,3], Daniela M. Bittencourt[2,3], Stevens K. Rehen[6,7], Martín H. Bonamino[8,9], Elibio Rech[2,3]

[1]Department of Cell Biology, Institute of Biological Science, University of Brasília, Brasília, Distrito Federal, Brazil;
[2]National Institute of Science and Technology in Synthetic Biology (INCT BioSyn), Brasília, Distrito Federal, Brazil;
[3]Embrapa Genetic Resources and Biotechnology, Brasília, Distrito Federal, Brazil;
[4]Center for Translational Research in Oncology, Instituto do Câncer do Estado de São Paulo, Hospital das Clínicas da Faculdade de Medicina de Universidade de São Paulo, São Paulo, São Paulo, Brazil;
[5]Molecular Carcinogenesis Program, Research Coordination, National Cancer Institute (INCA), Rio de Janeiro, Rio de Janeiro, Brazil;
[6]D’Or Institute for Research and Education (IDOR), Rio de Janeiro, Rio de Janeiro, Brazil;
[7]Institute of Biomedical Sciences, Federal University of Rio de Janeiro, Rio de Janeiro, Rio de Janeiro, Brazil;
[8]Cell and Gene Therapy Program, Research Coordination, National Cancer Institute (INCA), Rio de Janeiro, Rio de Janeiro, Brazil;
[9]Vice-Presidency of Research and Biological Collections (VPPCB), FIOCRUZ – Oswaldo Cruz Foundation Institute, Rio de Janeiro, Rio de Janeiro, Brazil

Elibio Rech: corresponding author: elibio.rech@embrapa.br;

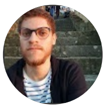

Marco Oliveira

Protocol Info: Marco A. de Oliveira, Lilian H. Florentino, Thais T. Sales, Rayane N. Lima, Luciana R. C. Barros, Cintia G. Limia, Mariana S. M. Almeida, Maria L. Robledo, Leila M. G. Barros, Eduardo O. Melo, Daniela M. Bittencourt, Stevens K. Rehen, Martín H. Bonamino, Elibio Rech . Protocol for assembly of a serine integrase-based platform for functional validation of genetic switch controllers in eukaryotic cells-Plant. **protocols.io** https://protocols.io/view/protocol-for-assembly-of-a-serine-integrase-based-c6rxzd7n

Created: Dec 20, 2023

Last Modified: Dec 23, 2023

PROTOCOL integer ID: 92695

ABSTRACT

This protocol details the assembly of a serine integrase-based platform for functional validation of genetic switch controllers in eukaryotic cells in plant.

ATTACHMENTS

pbt9ca8tp.docx

GUIDELINES

Troubleshooting:

TABLE 6. Troubleshooting for the Plant system stages

A	B	C	D
**Step**	**Problem**	Possible reason	**Solution**
1	Early appearance of inflorescences	A long photoperiod	Keep a photoperiod of up to 12 hours of light
1	Fungus-contaminated leaves	Fungi introduction via contaminated vessels, air, soil or water.<br>Excess moisture.	Plants should only be irrigated with clean distilled water.<br>Control air humidity and avoid damp soil.<br>Readily dispose of symptomatic plants.<br>For persistent events, apply fungicidal agents in soil.
1	Small, distorted, yellowish leaves	Nutritional deficiency	Use a good source of soil.<br>If necessary, fertilizer should be enriched with macro- and micronutrients.<br>Add 1X MS medium solution for the cultivation of Arabidopsis.
1	Purplish leaves	Excessive light exposure.	Adjust for proper photoperiod and light source intensity.
1	Presence of fungi and algae in soil and/or trays	Excess of water or contaminated soil.	Water only when necessary to control humidity in the pots (once every two days must be sufficient).<br>Autoclave soil mixture before planting
1	Presence of soil flies/ dark flies (Sciaridae)	Accumulation of organic matter	Remove excess water from the pots, and eliminate algae, slime and fungi that grow on the soil.<br>Install yellow adhesive traps just above plants (replace weekly).
1	Presence of white flies or aphids	Accumulation of organic matter	Apply a 0.03% solution of DECIS 25E. to prevent and treat !CAUTION Hazardous. Use PEP. Install yellow adhesive traps just above plants (replace weekly).

A	B	C	D
7	Leaves do not digest	Slow infiltration of enzymatic cocktail in plant tissue.	Incubate for one additional hour
7	Leaves still not digested	Old enzymatic solution.<br>Old or sick plants.	Discard the material and:<br>Prepare a new batch of enzymatic solution.<br>Check age and overall health of plants.
8	Solution retained in the mesh	Accumulation of leaf debris in the mesh	Use a 1 mL micropipette with a cut tip to press out any remaining enzyme solution from the sample.<br>If necessary, pipette up to 2 mL of cold W5 solution to wash the mesh.

MATERIALS

Biological materials

Arabidopsis thaliana plants at 60 days after germination.

▲CRITICAL The age of the plant is essential for good results in the isolation of protoplasts. Older plants produce a lower quality and quantity of protoplast.

Reagents

Plant Grow

- Substrate for greenery (Carolina Soil, Bioplant, PlantMax or equivalent)
- DECIS 25 EC® (Bayer, Active principle: deltamethrin 25 g.L-1)
- 0.5 X MS liquid medium (see "Reagent Setup") when necessary for fertilization

Protoplast Isolation

- MES or 2-(N-Morpholino)ethanesulfonic acid **Merck MilliporeSigma (Sigma-Aldrich) Catalog #M3671**
- Bovine Serum Albumin (BSA) **Merck MilliporeSigma (Sigma-Aldrich) Catalog #A7906**
- Calcium chloride, CaCl2 (Sigma-Aldrich, cat. no. C2661 or equivalent)
- Cellulase from Trichoderma sp. **Merck MilliporeSigma (Sigma-Aldrich) Catalog #C1794**
- Driselase™ from Basidiomycetes sp. **Merck MilliporeSigma (Sigma-Aldrich) Catalog #D9515**
- Ethanol 70% (JFeres, cat. no. 50731 or equivalent)

- Magnesium chloride, $MgCl_2$ (Vetec, cat. no. V000149 or equivalent)
- D-Mannitol **Merck MilliporeSigma (Sigma-Aldrich) Catalog #M1902**
- Pectolyase from Aspergillus japonicus **Merck MilliporeSigma (Sigma-Aldrich) Catalog #P5936**
- Potassium Chloride **Merck MilliporeSigma (Sigma-Aldrich) Catalog #P9541**
- Potassium hydroxide, KOH (J.T.Baker, cat. no. 3140-19 or equivalent)
- Sodium chloride **Merck MilliporeSigma (Sigma-Aldrich) Catalog #S3014**
- Distilled water, dH_2O, sterile
- Ice

Protoplast transfection

- 1 $\mu g.\mu L^{-1}$ plasmid DNA
- MES hydrate **Merck MilliporeSigma (Sigma-Aldrich) Catalog #M8250**
- Calcium chloride **Merck MilliporeSigma (Sigma-Aldrich) Catalog #C2661**
- Distilled water, dH_2O, sterile
- D-Mannitol **Merck MilliporeSigma (Sigma-Aldrich) Catalog #M1902**
- PEG-4000 **Merck MilliporeSigma (Sigma-Aldrich) Catalog #81240**
- Potassium Chloride **Merck MilliporeSigma (Sigma-Aldrich) Catalog #P9541**
- Potassium hydroxide, KOH (J.T.Baker, cat. no. 3140-19 or equivalent)
- Ice

Protoplast Flow Cytometry

- COULTER CLENZ AGENT (500ML) **Beckman Coulter Catalog #8546929**
- Debubbler solution: Isopropyl alcohol 70% (Fisher Scientific, cat. no. A459)
- Flow Sight Calibration Beads (Amnis, cat. no. 400300)
- Rinse: Milli-Q deionized water, at least 0.22 µm filtered
- Phosphate buffered saline **Merck MilliporeSigma (Sigma-Aldrich) Catalog #P4417-50TAB**
- Sterilizer solution: 0.4-0.7% sodium hypochlorite (VWR, cat. no. JT9416-1)

Protoplast Viability assay

- Acetone **Merck MilliporeSigma (Sigma-Aldrich) Catalog #904082**
- Aluminum foil UV clean (any brand)
- DMSO (dimethyl sulfoxide) **Merck MilliporeSigma (Sigma-Aldrich) Catalog #D8418**
- Fluorescein Diacetate **Merck MilliporeSigma (Sigma-Aldrich) Catalog #F7378**

Plasmid extraction and Cloning

- Agarose **Merck MilliporeSigma (Sigma-Aldrich) Catalog #A9539**
- Ampicillin sodium salt **Merck MilliporeSigma (Sigma-Aldrich) Catalog #A9518**
- Distilled water, dH2O, sterile
- DNeasy Plant Mini Kit **Qiagen Catalog #69104/69106**
- dNTP set 100 mM **Invitrogen - Thermo Fisher Catalog #10297018**
- EcoRI - 10,000 units **New England Biolabs Catalog #R0101S**
- Kanamycin monosulfate **Merck MilliporeSigma (Sigma-Aldrich) Catalog #K1377**
- LB Broth (Lennox) **Merck MilliporeSigma (Sigma-Aldrich) Catalog #L3022**
- One Shot™ MAX Efficiency™ DH10B T1 Phage-Resistant Cells **Thermo Fisher Catalog #12331013**
- pGEM(R)-T Easy Vector System I **Promega Catalog #A1360**
- Platinum™ Taq DNA Polymerase **Thermo Fisher Catalog #10966018**
- QIAGEN Plasmid Maxi Kit (25) **Qiagen Catalog #12163**
- SYBR SAFE DNA stain **Invitrogen - Thermo Fisher Catalog #S33102**
- Wizard(R) Plus SV Miniprep DNA Purif. Sys., 50 preps w/Adp. **Promega Catalog #A1340**
- Wizard(R) SV Gel and PCR Clean-up System, 250 preps **Promega Catalog #A9282**
- XL1-Blue Supercompetent Cells **Agilent Technologies Catalog #200236**

Equipment

- Autoclave Vitale Class CD54 (Cristofoli or equivalent)
- Axiovert 135M fluorescence microscope (Carl Zeiss)
- Centrifuge MiniSpin® (Eppendorf, cat. no. 5452000816 or equivalent);
- Centrifuge 5804R, refrigerated (Eppendorf, cat. no. EP022628146) with Rotor A-4-81, with 15-30 mL buckets and adapters
- Ice Machine Super Ice (BenMax cat. no. BMGX26-07 or equivalent)
- Incubator Shaker Series I26 (New Brunswick Scientific)
- Laminar Flow Cabinet Sentinel Gold (ESCO cat. no. LHG-6CG-F8 or equivalent)
- Midi Plus-1 Horizontal Electrophoresis System (MajorScience cat. no. ME10-7-10 or equivalent)
- Millipore Milli-Q® Direct 8 Water Purification System (Marck ct. no. C85358)
- Thermomixer Confort (Eppendorf cat. no. EXT 20441 or equivalent)
- Water bath (Ultronic, model Q3.0/040A or equivalent)
- Vacuum chamber - Biobalistic Particle Delivery System PDS-1000/He System (Bio-Rad cat. no. 1652257 or equivalent)

Protoplast transfection

- Eppendorf® Centrifuge 5804R, refrigerated (Eppendorf, cat. no. EP022628146) with Rotor A-4-81, with 15-30 mL buckets and adapters
- Plate incubator (TECNAL, model TE-420 or equivalent)
- Whatman® UNIFLO® 25 syringe filters (Whatman cat. no. WHA9913-2502)

Flow cytometry

- Amnis® brand FlowSight® Imaging Flow Cytometer (Merck Millipore, cat. no. 100300 or equivalent)
- Eppendorf® Centrifuge 5804/5804R, refrigerated (Eppendorf, cat. no. EP022628146) with Rotor A-4-81, with 15-30 mL buckets and adapters

Solutions, Plasmid extraction and Cloning

- Centrifuge MiniSpin® (Eppendorf, cat. no. 5452000816)
- Gel Documentation System SmartView Pro 1100 Imager System, (Major Science, cat. no.UVCI-1100 or equivalent)
- Plate incubator (TECNAL, model TE-420 or equivalent)
- Magnetic stirrer with hot plate (any brand)
- NanoDrop 2000C Spectrophotometer (Thermo Scientific)

Equipment	
**PowerPac™ Basic Power Supply**	NAME
Basic Power Supply	TYPE
PowerPac	BRAND
1645050	SKU
https://www.bio-rad.com/en-uk/sku/1645050-powerpac-basic-power-supply?ID=1645050	LINK

- Precision scale (any brand)
- Incubator Shaker Series I26 (New Brunswick Scientific)
- Stir bars (any brand)
- scalpel (any brand)
- Surgical Blade nº 11 (any brand)

Equipment	
**T100™ Thermal Cycler**	NAME
Thermal Cycler	TYPE
Bio-Rad	BRAND
1861096	SKU
https://www.bio-rad.com/en-at/product/t100-thermal-cycler?ID=LZJU45E8Z	LINK

Glassware and disposable instruments

- 150 mL plastic cup (any brand)
- 10 µl pipette tips (any brand)
- 100 mL volumetric flask (any brand)
- 1 mL pipette tips WB (Axygen, cat. no.T-1005-WB-C or equivalent)
- 200 µl pipette tips WB (Axygen, cat. no.T-205-WB-C or equivalent)

Equipment	
**PIPETMAN P2, 0.2-2 µL, Metal Ejector**	NAME
Metal Ejector	TYPE
Gilson	BRAND
F144054M	SKU

Equipment	
**PIPETMAN P10, 1-10 µL, Metal Ejector**	NAME
Metal Ejector	TYPE
Gilson	BRAND
F144055M	SKU
https://gb.gilson.com/GBSV/pipetman-p10-1-10-micro-l-metal-ejector.html	LINK

Equipment	
**PIPETMAN P100, 10-100 µL, Metal Ejector**	NAME
Metal Ejector	TYPE
Gilson	BRAND
F144057M	SKU
https://gb.gilson.com/GBSV/pipetman-p100-10-100-mi	LINK

Equipment	
**PIPETMAN P1000, 100-1000 µL, Metal Ejector**	NAME
Metal Ejector	TYPE
Gilson	BRAND
F144059M	SKU
https://gb.gilson.com/GBSV/pipetman-p1000-100-1000-micro-l-metal-ejector.html	LINK

Equipment	
**PIPETMAN P5000, 500-5000 µL**	NAME
Micropipette	TYPE
Gilson	BRAND
F144066	SKU
https://gb.gilson.com/GBSV/pipetman-p5000-500-5000-micro-l.html	LINK

- 44-µm metal mesh (any brand)
- 500 mL volumetric flask (any brand)
- Beaker, 1000 mL, 500 mL 100 mL, 50 mL (any brand)
- Cell culture plate, 12-wells, flat bottom (Kasvi, cat. no. K12-012 or equivalent)
- Conical centrifuge tube, 15 mL and 50 mL (Kasvi, cat. no. K19-0015 and K19-0050 respectively or equivalent)
- Round bottom Corex glass tubes, 15mL, 30 mL (no. 8441 and 8445 respectively, or equivalent)
- Glass bottle with a screw lid, 500 mL and 300 mL (any brand)
- Glass cell culture dishes ø90 mm and ø120 mm (any brand)
- Glass Coverslips (24 x 50 mm) (Olen, cat. no. K5-245)
- Glass Microscope Slides (26 x 76 mm) (Olen, cat. no. K5-710)
- Graduated syringe without needle 20 mL and 50 mL (any brand)
- Graduated cylinder, 100 mL, 1000 mL (any brand)
- Membrane Filter, 0.22 µm pore size (KASVI, cat. no. K18-230 or equivalent)

- Membrane Filter, 0.45 µm pore size (KASVI, cat. no. K18-430 or equivalent)
- Metal spatula (any brand)
- Metal tweezer (any brand)
- Microtubes, 1.5 mL (Axygen Scientific, cat. no. MCT -150)
- PCR tubes 0.2 mL (Axygen Scientific, cat. no. PCR-02-C or equivalent)
- Plant vessels (any brand)
- Steritop® vacuum filtration system 0.22 µm (Millipore, cat. no.SCGPS05RE)
- Whatman® UNIFLO® 25 syringe filters (Whatman cat. no. WHA9913-2502)

Reagent Setup

Prepare stock solutions (Tables X1-X3) in advance and follow the instructions outlined below.
▲CRITICAL The volumetric flask is the most suitable glassware for preparing solutions. It is used to measure accurate amounts of liquid. Try to use a volumetric flask whenever possible; otherwise, use a graduated cylinder. After preparing the solution and adjusting to the final volume, transfer the solution to a beaker and filter or transfer the solution to a glass bottle with a screw lid to autoclave it.
! CAUTION Do not screw the glass bottle lid on completely as it may explode inside the autoclave. Store the solutions as recommended.

Protoplast Isolation

Stock Solutions

KOH 2.5 N

Use a 100 mL beaker. Carefully dissolve 7.01 g of KOH in approximately 30 mL of distilled water. Mix well, then adjust the final volume to 50 mL with dH2O. Transfer to a 50 mL glass bottle with a screw lid.
! CAUTION Hazardous. KOH is corrosive and irritant. Wear proper personal protective equipment (PPE).
▲CRITICAL KOH is a base and slowly dissolves glass. Store at 25 °C in a polycarbonate conical centrifuge tube for up to 2 years . Label it properly as corrosive

MES (pH 5,7) 0.1 M

Use a 250 mL beaker. Dissolve 3,91 g of MES in approximately 150 mL of distilled water. Add a stir bar and put in a magnetic stirrer. Adjust pH with KOH to pH 5,7. Mix well, then adjust the final volume to 200 mL with dH2O. Filter it with Steritop® vacuum filtration system 0.22 µm in a 500 mL sterile glass bottle with screw lid. Label it properly. Store at 25 °C for up to 3 months.

Mannitol 0.8 M

Use a 500 mL beaker. Dissolve 58,30 g of mannitol in approximately 300 mL of distilled water. Mix well, then adjust the final volume to 400 mL with dH2O and filter it with a Steritop® vacuum filtration system 0.22 µm in a 500 mL sterile glass bottle with a screw lid. Label it properly. Store at 25 °C for up to 3 months.
▲CRITICAL Mannitol shows low solubility at this concentration. Gently pour under constant stirring until the complete dissolution.

NaCl 5 M

Use a 250 mL beaker. Dissolve 58.44 g of NaCl in approximately 150 mL of distilled water. Mix well, then adjust the final volume to 200 mL with dH2O and filter it with a Steritop® vacuum filtration system 0.22 µm in a 250 mL sterile glass bottle with a screw lid. Label it properly. Store at 25 °C for up to 3 months.

MgCl2 1 M

Use a 100 mL beaker. Dissolve 4.76 g of MgCl2 in approximately 30 mL of distilled water. Mix well, then adjust the final volume to 50 mL with dH2O and filter it by using a 50 mL syringe with a 0.22 µm filter in a 50 mL sterile glass bottle with a screw lid. Label it properly. Store at 25 °C for up to 3 months.

CaCl2 1 M

Use a 250 mL beaker. Dissolve 22.2 g of CaCl2 in approximately 150 mL of distilled water. Mix well, then adjust the final volume to 200 mL with dH2O and filter it with a Steritop® vacuum filtration system 0.22 µm in a 250 mL sterile glass bottle with a screw lid. Label it properly. Store at 25 °C for up to 3 months.

KCl 1 M

Use a 100 mL beaker. Dissolve 3.73 g of KCl in approximately 30 mL of distilled water. Mix well, then adjust the final volume to 50 mL with dH2O and filter it by using a 50 mL syringe with a 0.22 µm filter in a 50 mL sterile glass bottle with a screw lid. Label it properly. Store at 25 °C for up to 3 months.

W5 Solution

Use a 500 mL beaker. Mix 12.32 mL of 5 M NaCl, 50 mL of 1 M CaCl2, 2 mL of 1 M KCl, and 8 mL of 0.1 M MES (pH 5.7). Adjust the final volume to 400 mL with dH2O and filter it with a Steritop® vacuum filtration system 0.22 µm in a 500 mL sterile glass bottle with a screw lid. Label it properly. Store at 4 °C for up to 3 months.

Enzymatic Solution

Step 1

Use a 200 mL beaker. Mix 62.5 mL of 0.8 M mannitol, 2 mL of 1 M KCl and 20 mL of 0.1 M MES (pH 5.7), and then dissolve 1.5 g of cellulase, 0.5 g of Driselase and 0.2 g of Pectolyase.
▲CRITICAL Stir slowly with heat at 55 °C for 10 minutes.
▲CRITICAL Cool the solution before proceeding to step 2.

Step 2

Add 100 mg of BSA and 1 mL of 1 M CaCl2 and mix gently. Adjust the final volume to 100 mL with dH2O and filter it by using a 50 mL syringe with a 0.22 µm filter.
▲CRITICAL Divide into 10 mL aliquots in 15 mL conical tubes. Label it properly. Store at -20 °C for up to 3 months.

MMg Solution

Use a 50 mL beaker. Add 25 mL of 0.8 M mannitol, 0.75 mL of 1 M MgCl2 and 2 mL of 0.1 M MES (pH 5.7). Adjust the final volume to 50 mL with dH2O. Filter it by using a 50 mL syringe with a 0.22 µm filter in a glass bottle with a screw lid. Store at 4 °C. Label it properly.

Working Solutions (reagents and respective final concentrations are listed in Table 2)

A	B	C
Component	Stock solution	Final concentration
W5 Solution		
NaCl	5 M	154 mM
CaCl2	1 M	125 mM
KCl	1 M	5 mM
MES (pH 5.7)*	0.1 M	2 mM
Enzymatic Solution		
Mannitol	0.8 M	500 mM
KCl	1 M	20 mM
MES (pH 5.7)*	0.1 M	2 mM
Cellulase		1.5% wt/vol
Driselase		0.5% wt/vol
Pectolyase		0.2% wt/vol
CaCl2	1 M	20 mM

A	B	C
BSA		1 mg/mL
MMg Solution		
Mannitol	0.8 M	400 mM
MgCl2	1 M	15
MES (pH 5.7)*	0.1 M	4 mM

* Use a KOH solution to adjust the MES stock solution pH.

Protoplast transfection (reagents and respective final concentrations are listed in Table 3)

40% PEG Solution

Use a 50 mL conical centrifuge tube. Dissolve 8 g of PEG4000 in 10 mL of dH2O. Add 5 mL of 0.8 M mannitol and 2 mL of 1 M CaCl2. Adjust the final volume to 20 mL with dH2O and mix gently.
▲CRITICAL Prepare fresh and cut off sterile 1 mL plastic tips to pipette it. Do not store the 40% PEG solution. Label it properly.

WI Solution

Use a 100 mL beaker. Add 31.25 mL of 0.8 M mannitol, 1 mL of 1 M KCl and 2 mL of 0.1 M MES (pH 5.7). Adjust the final volume to 50 mL with dH2O. Filter it by using a 50 mL syringe with 0.22 µm in a 50 mL glass bottle with screw lid. Label it properly. Store at 4 °C for up to 3 months.

Table 3. Reagents and final concentration for protoplast transfection working solutions.

A	B	C
Component	Stock solution	Final concentration
40% PEG Solution		
PEG4000		40% wt/vol
Mannitol	0.8 M	200 mM
CaCl2	1 M	100 mM
W1 Solution		
Mannitol	0.8 M	500 mM

A	B	C
KCl	1 M	20 mM
MES (pH 5.7)*	0.1 M	4 mM

* Use a KOH solution to adjust the MES stock solution pH.

Viability assay (reagents and respective final concentrations are listed in Table 4)

Fluorescein diacetate (FDA) solution

Prepare a stock solution by dissolving 5 mg of FDA in 1 mL of acetone. Store at 4 °C for up to 7 days. Label it properly.

! CAUTION Hazardous. Acetone is toxic, flammable and irritating. Wear proper personal protective equipment (PPE). Add 10 µL of FDA stock solution in 2.5 mL of W1 solution.
▲CRITICAL Prepare fresh and cover with aluminum foil.

Table 4. Reagents and final concentration for FDA viability assay working solutions

A	B	C
Component	Stock solution	Final concentration
Fluorescein diacetate (FDA) Solution		
FDA	5 mg.mL-1	20 µL.mL-1

Plant growth ● Timing 4-6 weeks

1 Grow Arabidopsis thaliana ecotype Columbia plants in a 150 mL plastic cup with aerated, moist, fertilized and autoclaved soil in an environmentally controlled chamber with a medium photoperiod (12 h light/12 h dark at 22 °C) under low light (optimum light is approximately 150 µE.m-2.s-1) and 50-60% relative humidity.

Note

▲CRITICAL STEP Pierce the base of the plastic cup to drain excess water and prevent root rot.
▲CRITICAL STEP Always maintain seedling production. Remember to transplant new plants every 2 weeks to ensure that the experiments can be repeated. Label each batch of plants properly.
▲CRITICAL STEP Due to laboratory logistics, we used a photoperiod of 12 h light/12 h dark, but the recommended photoperiod condition to optimize the production of the plant's vegetative area is 8 h light/16 h dark.
? TROUBLESHOOTING

Protoplast isolation. ● Timing 4-6 hours

6h

2 Choose two to four healthy plants and take 20 leaves (3-4 cm long) by cutting each petiole vertically with the help of a scalpel.

Note

! CAUTION Scalpel blade is sharp: use it with extreme caution.
▲CRITICAL STEP Leaf selection will directly impact protoplast production. Watch the development of the plant aerial part.
▲CRITICAL STEP Place the leaves immediately in a 100 mL beaker with 50 mL of sterile distilled water. This step is necessary to I) prevent the formation of air bubbles in the leaf, which could hamper the infiltration of the enzyme solution, and II) wash away any remnants of soil and dust from the leaves.

3 (Optional) Sterilize the leaves by washing once in 70% ethanol, for 00:05:00 in a 1% sodium hypochlorite solution, and five times in sterile dH_2O. 5m

Note

! CAUTION Sodium hypochlorite is hazardous: wear proper personal protective equipment (PPE).

4 Using a metal tweezer, transfer one leaf with the adaxial side facing upward to a glass cell culture dish (90 mm x 15 mm) containing 25 mL of the W5 solution. Using the scalpel, extract and discard the remaining petiole and make sequential cross-sections (1-2 mm thick) from the midrib to the leaf margin. It is not necessary to cut the leaves completely, only to make little “scratches” on the leaf surface.

Note

! CAUTION Scalpel blade is sharp, use it with extreme caution.
▲CRITICAL STEP Leaf cuts are made in W5 solution to prevent air bubble formation in the leaf, which could hamper the infiltration of the enzyme solution, thus influencing the achievement of a satisfactory number of viable protoplasts.

5 Using a metal tweezer, carefully pass the chopped leaf on the cell culture dish´s edge to remove excess W5 solution and transfer the leaf with the adaxial side down to another glass cell culture dish (60 mm x 15 mm) containing 5 mL of the enzyme solution.

Note

▲CRITICAL STEP Repeat steps 3 and 4 one leaf at a time.
▲CRITICAL STEP Place the aliquot of stock enzyme solution on ice to thaw slowly before use.

6 Transfer the cell culture dish with the enzyme solution and chopped leaves into the vacuum chamber and vacuum infiltrate 3 times for approximately 00:00:05 under 500 mm Hg pump pressure. 5s

Note

! CAUTION With each vacuum repetition, release the vacuum very carefully.
▲CRITICAL STEP Vacuum is essential for enzyme solution leaf infiltration. Vacuum until air bubbles emerge from the leaves.

7 Cover the cell culture dish with aluminum foil and then incubate in the platform shaker with gentle swirling (up to 40 rpm) for 03:00:00 at Room temperature . 3h

Note

▲CRITICAL STEP Digestion should be performed in the dark to prevent oxidation and to reduce the photosynthetic pathway.

8 After 3 hours of incubation, release protoplasts by swirling the cell culture dish for 1 minute or until the solution turns green.

Note

? TROUBLESHOOTING

9 Filter the digested sample through a 74-µm cell mesh and carefully transfer it into a 30 mL glass round bottom centrifugation tube.

Note

▲CRITICAL STEP Place bottle of W5 on ice thirty minutes before use (steps 5, 11, 13). ? TROUBLESHOOTING

10 Wash the mesh with up to 10 mL of ice-cold W5 solution to remove all remaining protoplasts.

11 Centrifuge the sample at 100 x g, 4°C, 00:02:00 . 2m

Note

▲CRITICAL STEP Use a refrigerated centrifuge for all centrifugations. Turn on and program the centrifuge to refrigerate 30 minutes before use.
▲CRITICAL STEP Use a swing-bucket rotor. The swing-type rotor allows the pellet to be positioned exactly at the bottom of the tube, which facilitates the discard of the supernatant. Set up the refrigerated centrifuge to slow acceleration and deceleration. Follow these critical instructions for steps 14, 16, 26, and 33 as well..

12 Carefully remove supernatant, leaving enough solution to cover the protoplasts (green pellet).

Note

▲CRITICAL STEP Use a 5000 µl pipette with a cut end tip to gently pipette out the supernatant. The pellet is very fragile. Also apply this method in steps 17 and 21.

13 Carefully resuspend the protoplasts in 20 mL of ice-cold W5 solution and gently rock the tube until the protoplasts are resuspended.

Note

▲CRITICAL STEP Do not resuspend the pellet by pipetting to prevent rupturing of intact protoplasts. Just gently swirling the tube. Follow these critical instructions in steps 15 and 18.

14 Centrifuge 100 x g, 4°C, 00:02:00 . Remove the supernatant with a 5000 µl pipette, again leaving enough to cover the protoplasts. 2m

15 Carefully resuspend protoplasts in 20 mL of ice-cold W5 solution and then incubate protoplasts On ice for 00:30:00 . 30m

Note

▲CRITICAL STEP Use this time to prepare the 40% PEG solution, as this can take some time to fully dissolve.

16 Swirling the protoplast tube gently until the protoplast pellet is completely resuspended and centrifug 100 x g, 4°C, 00:02:00 2m

17 Carefully remove the supernatant with a 5000 µl micropipette, again leaving enough to cover the protoplasts.

Note

▲CRITICAL STEP Place bottle of MMg solution on ice 30 minutes before use.

18 Resuspend the protoplasts to bring the total volume in the 30 mL tube to 1 mL with ice-cold MMg solution.

Note

▲CRITICAL STEP Use another tube with the same volume for comparison. Swirling gently to resuspend.

19 Count protoplasts and adjust the concentration to 4 x 10^5 protoplasts/mL with MMg solution (see steps 20-22).
Counting the protoplasts:

20 In a 1.5 mL microcentrifuge tube, dilute 5 µL of the protoplast solution obtained in step 18 above into 1 mL of MMg solution, then pipet 2 µL of the diluted protoplasts on a microscope slide and count the total number of protoplasts in that 2 µl drop.

Note

▲CRITICAL STEP Only count whole, circular protoplasts that have no cell wall remaining. Count from 5 different drops, one drop at a time to avoid inaccuracies and average them.

21 To dilute to 4 x 105 protoplasts/mL, apply the formula:

Final volume of MMg solution (mL)= (D_(A)× V_S×100)/(4×〖 10〗 ^5)

where DA is the average number of protoplasts from the five 2 µl drops and VS is the exact volume of resuspended protoplasts in microliters.

Note

▲CRITICAL STEP Measure the exact volume of resuspended protoplasts using a 5000 µl micropipette.

22 Add MMg solution to bring the original protoplast solution to the final volume calculated in the previous step.

Protoplast transformation. ● Timing 1-2 hours

23 In a 15 mL glass round bottom centrifugation tube, mix by gently swirling the protoplast solution, DNA solution, and 40% PEG solution, according to Table 5. Add the components in this order. Increase the

number of reactions as needed. Use one tube for each reaction.

Note

▲CRITICAL STEP Do not vortex, pipette up-and-down or invert tubes to mix the solution.
▲CRITICAL STEP Do not produce bubbles while mixing, which can cause protoplasts to explode.

Table 5 - Reaction mix for protoplast transformation

A	B	C
**Order**	**Component**	**Instructions**
01	100 µL of protoplast solution (concentration 4 x 105 protoplasts/mL)	Swirl the tube with protoplast solution gently and thoroughly to make sure that no pellet is formed. Use a wide-bore pipette tip to prevent damage to the protoplasts. Do not make bubbles.
02	10 µL of each plasmid DNA (concentration 1 µg x mL-1)	Use a filter pipette tip to prevent contamination.
03	110 µL 40% PEG solution	The solution is very thick; pipette very carefully. Gently swirl the tube until the solution is well mixed and layers can no longer be seen in the solution. Do not introduce bubbles.
320 µL final volume		The final volume will vary according to the number of plasmids used in the transformation (up to 5).

24 Incubate the 15 mL tubes for 00:15:00 at Room temperature. 15m

25 To stop the reaction, add 2 volumes of ice-cold W5 Solution. Gently swirl the tubes to mix.

26 Centrifuge at 100 x g, 4°C, 00:02:00. 2m

Note

▲CRITICAL STEP The pellet is very delicate: handle the tubes carefully.
▲CRITICAL STEP Place bottle of W1 solution on ice 30 minutes before use.

27 The supernatant was carefully removed, leaving enough solution to cover the protoplast.

28 Resuspend the protoplast in 500 µL of ice-cold W1 Solution. Gently swirling the tube to mix. This volume corresponds to one single replica transformation reaction.

Note

▲CRITICAL STEP Completely and carefully resuspend the pellet.

29 Transfer the protoplast solution to a 12-well cell culture plate. If you perform multiple transformation reactions, transfer each reaction to a single and labeled well of the plate.

Note

▲CRITICAL STEP If you are working with multiple reactions and/or plates, make sure that you labeled the wells/plates correctly.

30 Place the lid on the plate and seal with Parafilm. Set the plate in a wet chamber and incubate for 24 h in the dark under gentle shaking (up to 40 rpm) at 25 °C - 30 °C. To make a dark wet chamber, put some paper towels in a plastic box/tray, wet with distilled water, place the plates and cover with aluminum foil.

Note

▲CRITICAL STEP The wet chamber prevents the samples from trying to dry out.
▲CRITICAL STEP The plates should be maintained in the dark.
▲CRITICAL STEP The time of incubation is enough to observe the accumulation of GFP in these conditions. For other situations, different times of incubation should be tested.

31 After 24 h of incubation, put the sealed 12-well cell culture plate in an Axiovert 135 M fluorescence microscope under UV light with filter set 15 (Carl Zeiss). Excitation: BP 546; beam splitter: FT 580; emission: LP 590. Capture images of GFP emission with attached DS-Ri1 digital camera (Nikon).

Flow cytometry ● Timing 30 min by sample

1m

32 Transfer all of the well contents to a labeled 1.5 mL microcentrifuge tube.

Note

▲CRITICAL STEP Use a wide-bore 1000 µL pipette tip to prevent damage to the protoplasts.

33 Centrifuge at 100 x g, 4°C, 00:01:00 in a swing-bucket rotor for plates with 1.5-2 mL microcentrifu[illegible] 1m
tubes block adapter.

Note

▲CRITICAL STEP Do not use a microcentrifuge: the pellet will stay in the well of the microtube and prejudice the quality of the readings.

34 Carefully remove the supernatant, leaving approximately 50 µL of the solution.

35 Flick the tube gently to thoroughly resuspend the pellet.

Note

▲CRITICAL STEP Carefully resuspend the pellet thoroughly. Prevent bubbles from forming in the bottom of the tube.

36 Analyze the presence and intensity of GFP fluorescence in the Amnis® brand FlowSight® Imaging Flow Cytometer (see Equipment Setup).

37 Repeat steps 32-36 for each well/sample.
