# Supplementary material for: Protocol for the establishment of a serine integrase-based platform for functional validation of genetic switch controllers in eukaryotic cells
Source: PLoS One. 2024 May 23;19(5):e0303999. doi: 10.1371/journal.pone.0303999 (PMC11115199; doi:10.1371/journal.pone.0303999)
Supplement: S5 File — (PDF) [file pone.0303999.s005.pdf]

MOLECULAR ANALYSES

In 1 collection

Marco A. de Oliveira[1,2], Lilian H. Florentino[1,2,3], Thais T. Sales[1,2,3], Rayane N. Lima[2,3], Luciana R. C. Barros[4], Cintia G. Limia[5], Mariana S. M. Almeida[2,3], Maria L. Robledo[5], Leila M. G. Barros[2,3], Eduardo O. Melo[2,3], Daniela M. Bittencourt[2,3], Stevens K. Rehen[6,7], Martín H. Bonamino[8,9], Elibio Rech[2,3]

[1]Department of Cell Biology, Institute of Biological Science, University of Brasília, Brasília, Distrito Federal, Brazil;
[2]National Institute of Science and Technology in Synthetic Biology (INCT BioSyn), Brasília, Distrito Federal, Brazil;
[3]Embrapa Genetic Resources and Biotechnology, Brasília, Distrito Federal, Brazil;
[4]Center for Translational Research in Oncology, Instituto do Câncer do Estado de São Paulo, Hospital das Clínicas da Faculdade de Medicina de Universidade de São Paulo, São Paulo, São Paulo, Brazil;
[5]Molecular Carcinogenesis Program, Research Coordination, National Cancer Institute (INCA), Rio de Janeiro, Rio de Janeiro, Brazil;
[6]D'Or Institute for Research and Education (IDOR), Rio de Janeiro, Rio de Janeiro, Brazil;
[7]Institute of Biomedical Sciences, Federal University of Rio de Janeiro, Rio de Janeiro, Rio de Janeiro, Brazil;
[8]Cell and Gene Therapy Program, Research Coordination, National Cancer Institute (INCA), Rio de Janeiro, Rio de Janeiro, Brazil;
[9]Vice-Presidency of Research and Biological Collections (VPPCB), FIOCRUZ – Oswaldo Cruz Foundation Institute, Rio de Janeiro, Rio de Janeiro, Brazil

Elibio Rech: corresponding author: elibio.rech@embrapa.br;

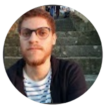

Marco Oliveira

ABSTRACT

This protocol details the molecular analyses of assembly of a serine integrase-based platform for functional validation of genetic switch controllers in eukaryotic cells

Protocol Info: Marco A. de Oliveira, Lilian H. Florentino, Thais T. Sales, Rayane N. Lima, Luciana R. C. Barros, Cintia G. Limia, Mariana S. M. Almeida, Maria L. Robledo, Leila M. G. Barros, Eduardo O. Melo, Daniela M. Bittencourt, Stevens K. Rehen, Martín H. Bonamino, Elibio Rech . MOLECULAR ANALYSES. **protocols.io** https://protocols.io/view/molecular-analyses-c6rzzd76

Created: Dec 20, 2023

Last Modified: Dec 21, 2023

PROTOCOL integer ID: 92697

ATTACHMENTS

pbt9ca8tp.docx

Primer design ● Timing 1d

1 Select approximately 20 nucleotides both upstream and downstream of the core region of each att site formed in the reporter plasmid after recombination takes place.

Note

▲CRITICAL STEP forward primer must anneal to attL, while the reverse primer will anneal to the attR sequence.

2 Use an online oligo design tool to define the best forward primers annealing to promoter sequence and reverse primers annealing to terminator sequence present in the reporter plasmid.

3 Define oligo pairs to obtain two amplicons for each reporter plasmid.

Note

▲CRITICAL STEP: Primer pairs must consist of a forward oligo annealing to the attL site and a reverse oligo annealing to the terminator region for sequencing proper attR site formation in amplicon I and a forward oligo annealing to the promoter region and a reverse oligo annealing to the attR site for sequencing proper attL site formation in amplicon II. (Figure 5).

The primers used in our studies are presented in **Table 7**.

A	B	C	D
Oligonucleotides used for amplification of Amplicon I and sequencing of attL sites			
**Promoter**	Forward primer (5’ -> 3’)	**nt**	**Model**
EFa_966F	TTCTCGAGCTTTTGGA GTACGTCGTCTTTAGG TTG	35	Mammal
35S_282F	ATTGATGTGATATCTCC ACTGACGTAAGGGATG ACGCAC	39	Plant
**attR**	Reverse primer (5’-> 3’)	**nt**	**Model**

A	B	C	D
attR _Int2_R	GTGTCTACGCGAGATT CTCGCCGGACCGTCGA CATACTGC	40	All models used
attR _Int4_R	AGTTTTCAACCCTTGAT TTGAATAAGACTGCTG CTTGTGT	40	
attR _Int5_R	ATAACTCTCCTGGGAG CGCTACACGCTGTGGC TG	34	
attR _Int7_R	CTGTGTGAGAGTTAAG TTTACATGGGCAAAGT TGATGAC	39	
attR _Int9_R	TGGAAGTGTGTATCAG GTAACTGGATACCTCA TC	34	
attR _Int13_R	GTAGAACTTGACCAGT TGGTCCTGTAAATATA AGCAATCC	40	
attR _phiC_R	CCAACTGGGGTAACCT TTGGGCTCC	25	
attR _Bxb1_R	CTGGTCAACCACCGCG GTCTCCGTCGTCAGGA TC	34	
Oligonucleotides used for amplification of Amplicon II and sequencing of attR sites			
**attL**	**Forward primer (5'-> 3')**	**nt**	**Model**
attL_Int2_F	GGAGTAGCTCTTCGCC CGAGAACTTCTGCAAG	32	
attL_Int4_F	CGACCTGAAATTTGAA TTAGCGGTCAAATAAT TTGTA	37	
attL_Int5_F	GACGGCCTGGGAGCGT TGACAACTTGCGCACC	32	

A	B	C	D
attL_Int7_F	GTCCGTCTGGGTCAGTTGCCTAACCTTAACTTTTAC	36	All models used
attL_Int9_F	ATAATTGGCGAACGAGGTATCTGCATAGTTATTCCGAAC	39	
attL_Int13_F	TCCAGATCCAGTTGTTTTAGTAACATAAATACA	33	
attL_phiC_F	TGCCAGGGCGTGCCCTTGAGTTCTCTCAGT	30	
attL_Bxb1_F	TGTCGACGACGGCGGTCTCAGTGGTGTACGGT	32	
**Terminator**	**Reverse primer (5' -> 3')**	**nt**	**Model**
TermiAni_205R	AATGATTTGCCCTCCCATATGTCCTTCCGAGTG	33	Mammal
NOSt_283R	ATAACAATTTCACACAGGAAACAGCTATGACATGATTACG	40	Plant

Target sequence amplification by PCR ● Timing 5h

4 Use a high-fidelity polymerase with non-template–dependent terminal transferase activity to insert a deoxyadenosine and the ends of generated amplicons.

Note

▲CRITICAL STEP Amplicon modification is important for cloning into pGEM-t-Easy to be sequenced.

5 Prepare a PCR mix for all reactions plus one (n+1) to account for pipetting errors. Include a negative control with water instead of DNA; positive control will require a previous synthesis of the expected recombined reporter plasmid.

6 Combine the reagents in the order shown below in **Table 8,** mix well by vortexing and spin briefly:

TABLE 8. PCR reaction mix components

A	B
**Component**	Volume to add (µl)
**dH2O nuclease free**	**18.65**
**Buffer 10x**	**2.5**
**MgCl2 [50 mM]**	**1.5**
**PCR Fw primer [10 µM]**	**0.75**
**PCR Rev primer [10 µM]**	**0.75**
**dNTP [10 mM]**	**0.75**
**Taq DNA polymerase**	**0.1**

7 Add 24.5 µL of the PCR mix to 0.2 mL PCR tubes..

8 To each respective tube, add 20 ng of template DNA and adjust the final volume to 25 µl if the DNA is too concentrated.

Note

Negative controls were prepared first by adding an equivalent volume of nuclease-free water and closing lids before pipetting templates to minimize contamination risk.

9 Gently pipette each sample up and down ten times to mix thoroughly. Place the PCR microtubes into a thermal cycler, and run the following program listed in **Table 9** (volume = 25 µL).

TABLE 9. PCR cycling condition

A	B	C	D
Cycle no.	Denature	Anneal	Extend
1	94°C, 3min		
2-34	94°C, 30s	65°C, 30s	72°C, 60s
35			72°C, 5min

Note

▲CRITICAL STEP Given the need for primers to align to a defined att site sequence, some parameter adjustments, such as Tm, GC content and 3' end base composition, will be limited and can vary from one integrase reporter to another, requiring adjustments to PCR cycling conditions.

10 Resolve amplicons by electrophoresis in agarose gel following PCR. Run settings and gel density will depend on amplicon size according to the analyzed gene length and oligo pairs used.

Note

▲CRITICAL STEP Load the same negative control in the every gel both technical (PCR without DNA) and biological (PCR using DNA from groups transformed with only either reporter plasmid or integrase plasmid) to ensure obtained bands indicate DNA inversion by Integrase activity. ? TROUBLESHOOTING

Amplicon purification ● Timing 2d

11 Excise the amplicon bands by cutting a square around them with the help of a scalpel on a UV light or blue light transilluminator.

Note

▲CRITICAL STEP Use different scalpel blades for each band to avoid cross-contamination of samples. ! CAUTION UV light can damage DNA, nicking and possibly removing DNA strand ends and interfering with downstream cloning steps. When available, blue light is highly recommended. If using UV, proceed quickly, turning the transilluminator off after making the cuts in the gel.

12 Proceed with amplicon purification using commercial DNA Clean-Up and Concentration kits, following the manufacturer's recommendations.

13 Clone purified amplicons in an entry vector to ensure high-quality sequencing results. Although specifics may vary depending on the plasmid, we recommend a molar ratio of 1:3 (vector to amplicon) and 1.5 U of T4 ligase in 5 µl reactions with Overnight incubation at 16 °C.

14 DH10b chemically competent cells were transformed with ligation products.

Heatshock transformation of DH10b chemically competent cel...

2h 32m 45s

15 Add 5 µL of the ligation reaction to 200 µL of cells thawed On ice.

16 Incubate cells On ice for 00:30:00. 30m

17 Subject cells to heat shock at 42 °C for 00:00:45 and return to ice for 00:02:00. 2m 45s

18 Add 1 mL of LB or SOC medium.

19 Incubate at 37 °C for 01:00:00 and then plate different dilutions on LB plates with appropria 2h
selecting agents. Incubate Overnight at 37 °C.

20 Screening for positive transformants by colony PCR. A polymerase with less fidelity can be used in this step. Combine the reagents in the order listed in **Table 10** below, mix well by vortexing and spin briefly:
TABLE 10. PCR reaction mix components for colony screening.

A	B
**Component**	**Volume to add (µl)**
**dH2O nuclease free**	**18.65**
**Buffer 10x**	**2.5**
**MgCl2 [50 mM]**	**1.5**

A	B
**PCR Fw primer [10 µM]**	**0.75**
**PCR Rev primer [10 µM]**	**0.75**
**dNTP [10 mM]**	**0.75**
**Taq DNA polymerase**	**0.1**

21 Add 25 µL of the PCR mix to 0.2 mL PCR tubes.

22 With a sterile toothpick or 200 µl pipetting tip, pick approximately 1/3 of each colony and add it to their respective tubes containing the PCR mix.

23 Gently pipette each sample up and down ten times to mix thoroughly. Place the PCR microtubes into a thermal cycler, and run the following program listed in **Table 11** (volume = 25 µL)

TABLE 11. PCR cycling condition for colony screening

Cycle no.	Denature	Anneal	Extend
**1**	**94°C, 10min**		
**2-34**	**94°C, 30s**	**60°C, 30s**	**72°C, 90s**
**35**			**72°C, 5min**

24 Resolve amplicons by electrophoresis in agarose gel following PCR.

25 Select multiple confirmed clones to isolate plasmids using commercial kits following the manufacturer's recommendations and have the purified plasmids sequenced.

Note

▲CRITICAL STEP Have your samples sequenced in both directions and in replicates to check for sequencing errors and identify possible mutations and DNA damage resulting from integrase activity.

26 Analyze sequencing electropherograms and alignment to expected sequences to confirm proper DNA recombination by Integrase activity.

Note

Troubleshooting

TABLE 12. Troubleshooting for the molecular analyses stage

A	B	C	D
**Step**	**Problem**	**Possible reason**	**Solution**
10	Unspecific amplification and unexpected bands on agarose gel	Oligos annealing at att sites has a 3' end complementarity to both original and recombined att sites	Increase the annealing temperature to more sselective conditions
